# Social regression stems from a latent phase of GABAergic dysfunction and sensory breakdown in a model of synaptopathy

**DOI:** 10.1038/s41380-026-03751-9

**Published:** 2026-07-13

**Authors:** Lorenzo Ciano, Sebastian Sulis Sato, Filippo Galluzzi, Arianna Benedetti, Sine Mandrup Bertozzi, Alessandro Esposito, Francesca Franchi, Dmytro Shmal, Chiara Penzo, Martino Caramia, Elisabetta Colombo, Anna Fassio, Andrea Armirotti, Fabio Benfenati, Caterina Michetti

**Affiliations:** 1 Center for Synaptic Neuroscience and Technology, Istituto Italiano di Tecnologia, Largo Rosanna Benzi, 10, 16132 Genova, Italy; 2 Department of Experimental Medicine, Università degli Studi di Genova, Viale Benedetto XV, 3, 16132 Genova, Italy; 3 IRCCS Azienda Ospedaliera Metropolitana, Largo Rosanna Benzi, 10, 16132 Genova, Italy; 4 The Open University Affiliated Research Centre at Istituto Italiano di Tecnologia, Genova, Italy; 5 Analytical Chemistry Facility, Istituto Italiano di Tecnologia, Via Morego 30, 16163 Genova, Italy

**Keywords:** Neuroscience, Physiology

## Abstract

In autism spectrum disorder (ASD), children can exhibit a regressive phenotype with loss of previously acquired social and language skills in the first years of life. While the role of the GABAergic system in shaping neural circuits during development has been largely studied, its potential involvement in regressive phenotypes remains unclear. Here, we tracked the social and sensory development in *Synapsin2* knockout (*Syn2KO*) mice, a model of ASD characterized by defective inhibitory tonic current, social deficits, and epilepsy in adulthood. We show that, after a transient early phase of impaired vocalization, young (PND 30) *Syn2KO* mice reached a normal social behavior. However, social behavior was regressively lost in adult (PND 120) mice, with the absence of vocalizations during the male-female interaction test associated with a drastic degeneration of the preoptic area-periaqueductal gray vocal GABAergic circuit. This deterioration began at earlier stages but was behaviorally latent. Adult *Syn2KO* mice also displayed sensory dysfunctions and dysregulation of the GABAergic system in the integrative posterior parietal cortex, with hyper-responsiveness of bimodal light- and sound-sensitive neurons that paralleled the social decline. Boosting tonic inhibition from birth with chronic treatment with the GABA_A_ receptor agonist gaboxadol fully reversed social deficits, restored the integrity of vocal circuits, and the multimodal integration of sensory inputs. These results show that social regression in the *Syn2KO* model is a multiphase process characterized by early signs, a latent period of typical development, and a subsequent decline that can be reversed by enhancing tonic inhibition from development onward.

## Introduction

Brain development is a complex, temporally regulated process that begins with neuronal maturation, proceeds to the formation of synaptic contacts, and culminates in synaptic refinement and the stabilization of mature circuits [1]. The last period, called the critical period (CP), is characterized by enhanced plasticity and strong dependence on sensory experience and environmental influences [2]. Factors that impact synaptic function, plasticity, and sensory integration in early life can alter neurodevelopmental trajectories, leading to behavioral impairments and regression. Regression is defined as the loss of previously acquired socio-cognitive skills. Autism spectrum disorder (ASD) is the most frequent neurodevelopmental disorder in which regression occurs, usually targeting verbal communication [3–5]. Social deficits arise at different developmental stages and can manifest with early-onset, stagnation, or regressive patterns. This distinction depends on whether the phenotype starts in early life, interrupts an early stage of social development, or appears after a period of typical development. The regressive subtype is thought to be much more frequent than previously reported [6, 7]. The number of cases remains debated, although recent papers agree that both prevalence and incidence of regressive autism are around 30% [8–10]. In human patients, regression manifests with a substantial decline or loss of previously developed skills, with speech deficits as its most distinctive feature [10–12]. Data based on home videos and prospective studies in high-risk infants suggest that most of the children with regressive ASD also presented previous, subtle developmental alterations [9, 13, 14]. Thus, in at least a subset of cases, regression may reflect a multi-phasic process characterized by a mixed pattern of early delays, followed by a latent period of typical development and a subsequent decline [9, 15, 16].

Social interaction is a specific domain of higher-order cognitive functions and is heavily affected by regressive processes. It requires integration of the internal state with the real-time decisions of the social partner and the ability to exchange information in a context-dependent fashion [17]. It thus relies on the activation of multiple brain regions and network functions, which in turn depend on the proper maturation of sensory systems. It is well known that the experience-dependent maturation of inhibitory circuits drives the temporal dynamics of CP in sensory areas and is a fundamental prerequisite for proper brain development [18–20]. Since complex behaviors are integrated responses to sensory inputs, behavioral dysfunctions are widely thought to stem from impaired sensory integration. Indeed, several animal models of ASD display delayed maturation of inhibitory systems associated with dysfunctional sensory and multisensory integrations [21–23].

Comparative studies confirm that a precise balance between excitation and inhibition (E/I) is required for correct brain development, and E/I imbalances are associated with neurodevelopmental disorders [24–32]. In addition to GABAergic synaptic transmission, GABA spillover stimulates extrasynaptic GABA_A_ receptors (eGABA_A_Rs), generating the tonic GABA current that regulates intrinsic neuronal excitability and contributes to the proper brain maturation [33]. Given the dual role of tonic inhibition in controlling brain circuit development and excitability, eGABA_A_Rs are among the most studied pharmacological targets in neurodevelopmental disorders [34–36]. Defective tonic inhibition emerged as a common feature across several genetic models of ASD, including Fragile X, Angelman syndrome, and Rett syndrome [37–41]. Reduced inhibitory strength and impaired tonic inhibition, associated with hyperexcitability in several ASD models, may contribute to the frequent comorbidity between ASD and epilepsy [42–47].

Based on these considerations, essential relationships exist between GABA dysfunctions and the emergence of social deficits in ASD. However, the mechanisms by which an altered inhibitory tone dysregulates social circuits and behavioral skills remain unclear. Here, we investigated the role of GABAergic systems in multisensory integration and social abilities across development in the *Synapsin2* knockout (*Syn2KO*) mouse, a well-established model of ASD characterized by defective tonic GABA current, marked social deficits in the first days of life, and epilepsy in adulthood [48–53].

Despite the early signs, mice exhibited a typical social behavior at 1 month of age, followed by a severe regression at 4 months, associated with severe degeneration of the GABAergic vocal circuit and breakdown of sensory integration in the posterior parietal cortex (PPC). Moreover, the sustained enhancement of the GABA tonic current by the eGABA_A_R agonist gaboxadol (THIP) from birth to adulthood fully rescued social deficits, the integrity of vocal circuits, and multimodal sensory integration. These findings suggest that the *Syn2*KO model may be particularly relevant for investigating forms of regressive ASD characterized by subtle early developmental alterations preceding an apparently typical phase and subsequent decline. Using this framework, we identify tonic inhibition as a key mechanism supporting the maturation of sensory integration and social function.

## Results

### Loss of previously acquired socio-communicative skills in *Syn2KO* male mice

Interacting mice communicate with each other by means of social sniffing and ultrasound vocalizations (USVs) in a context-dependent fashion. Male-female interaction is the most characterized social context, with males exhibiting a clear courtship repertoire characterized by sniffing the female with the concomitant emission of USVs [54, 55]. Males acquire these attractive behaviors at ≈1 month of age, when sexual maturation is reached, and the CP is about to end [56]. We assessed the development of social skills by performing male-female social interaction tests in young (1-month-old, PND30) and adult (4-month-old, PND120) wild-type (WT) and *Syn2KO* mice.

**Fig. 1:**
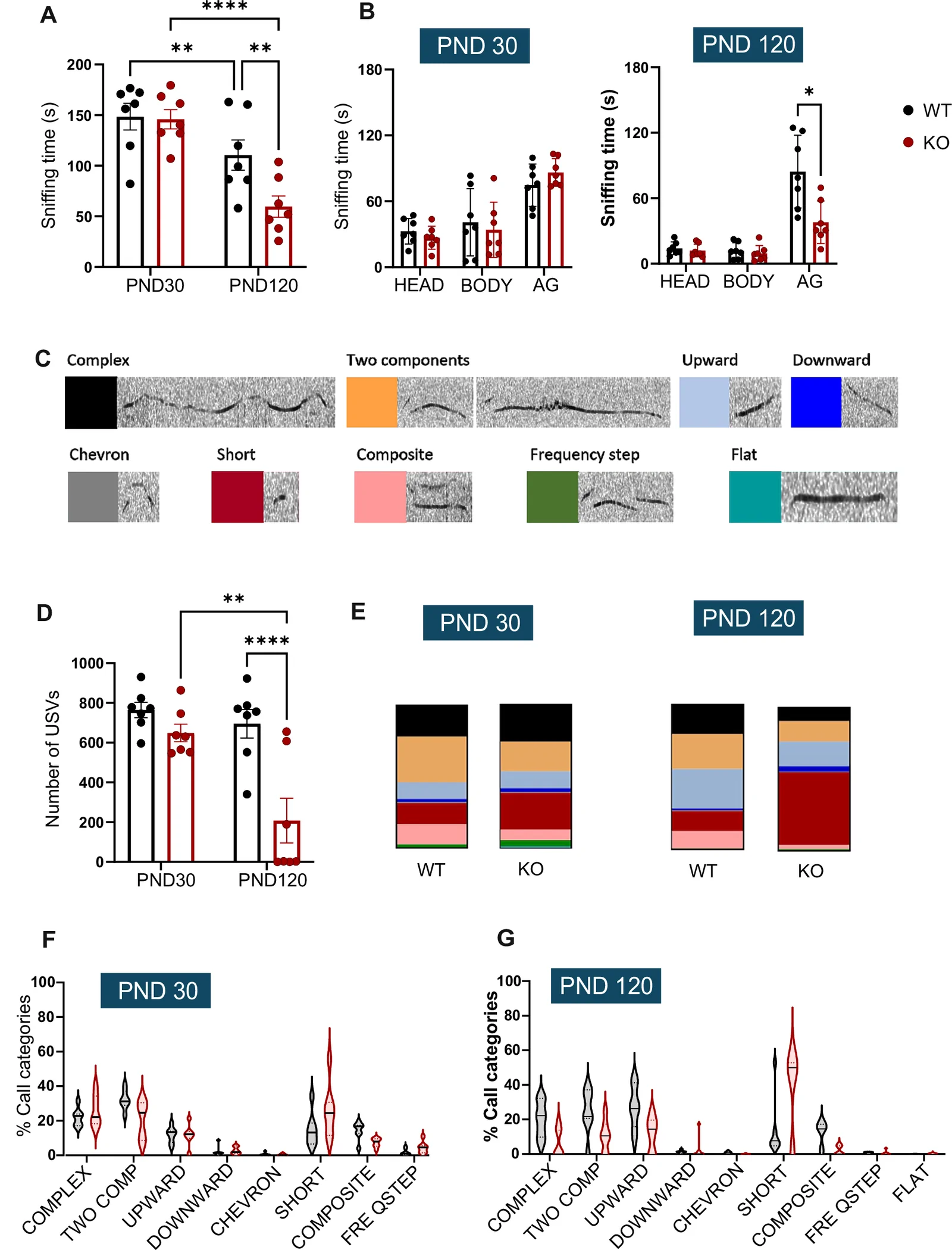
The *Syn2KO* male mouse recapitulates regressive social behavior in ASD. (**A**) Duration of sniffing behavior recorded during the male-female social interaction test in young (PND30) and adult (PND120) mice. The time spent in social exploration is expressed as cumulative time. **(B)** Time spent in social exploration of specific body regions (Head, Body, AnoGenital) at PND30 (*left*) and PND120 (*right*). (**C**) Representative syllables used for the characterization of USVs emitted during the male-female social encounter. **(D)** Total number of USVs emitted by male mice in interaction with an estrous female at PND30 and PND120. (**E**) Column graphs show the percentage of distinct syllables emitted by each genotype with respect to the total number of calls analyzed in each subject at PND30 (*left*) and PND120 (*right*). **(F,G)** Violin plots depict the distribution of call categories for each genotype at young (**F**; PND30) and adult (**G**; PND120) ages. Values are expressed in percentages and calculated as described in E. *Statistics*. Data are expressed as means ± SEM with superimposed individual experimental points (A,B,D). *p < 0.05; **p < 0.01; **** *p* < 0.0001 (N = 7 WT and 7 KO mice). (**A**) Cumulative Sniffing, RM-ANOVA. Time effect F_1,12_ = 75.02, *p* < 0.0001, ηp² = 0.86; Time × Genotype interaction: F_1,12_ = 11.37, *p* = 0.0056, ηp² = 0.49; Fisher’s LSD test: WT *vs* KO at PND120 *p* = 0.0071; WT-PND30 *vs* WT-PND120, *p* = 0.0028; KO-PND30 *vs* KO-PND120, *p* < 0.0001. **(B)** Sniffing body areas at PND30: RM-ANOVA. Body regions effect F_1.65, 19.8_ = 24.84, *p* < 0.0001, ηp² = 0.67; Genotype effect: F_1,12_ = 0.0048, *p* = 0.95, ηp² < 0.001; Body regions x Genotype interaction: F_2,24_ = 0.89, *p* = 0.42, ηp² = 0.07. Sniffing body areas at PND120: RM-ANOVA. Body regions effect F_1.047,12.56_ = 51.12, *p* < 0.0001, ηp² = 0.81; Genotype effect F_1,12_ = 7.76, *p* = 0.0165, ηp² = 0.36; Body regions x Genotype interaction: F_2,24_ = 10.23, *p* = 0.0006, ηp² = 0.46 - Bonferroni’s test: AG, WT *vs* KO *p* = 0.0103. **(D)** Number of USVs, RM-ANOVA. Time effect F_1,12_ = 11.99, *p* = 0.0047, ηp² = 0.50; Genotype effect F_1,12_ = 17.29, *p* = 0.0013, ηp² = 0.58; Time × Genotype interaction: F_1,12_ = 6.36, *p* = 0.0268, ηp² = 0.35; Fisher’s LSD test: WT *vs* KO at PND120 *p* < 0.0001; KO-PND30 *vs* KO-PND120, *p* = 0.0012. **(F)** Percent call categories: Mixed-model ANOVA. Call categories x Genotype interaction: F_8,108_ = 3.53, *p* = 0.0011, ηp² = 0.21 (Bonferroni’s test: not significant across genotypes). **(G)** Mixed-model ANOVA. Call categories x Genotype interaction: F_8,96_ = 5.43, *p* < 0.0001, ηp² = 0.31 (Bonferroni’s test: not significant across genotypes).

Animals tested at PND30 displayed intact socio-vocal behavior, as evidenced by both the cumulative time spent sniffing various body areas, including head, body, and the most explored anogenital (AG) area (Fig. 1A, B), and USVs (Fig. 1C–G). In contrast, the same group of *Syn2KO* mice tested in adulthood (PND120) displayed a strong impairment in both social (Fig. 1A, B; Supplementary Figure 1A) and vocal (Fig. 1C–G; Supplementary Figure 1B–D) abilities. Although the rate of social interaction decreased during development in WT animals, *Syn2KO* mice showed a marked impairment in both social interaction and communication at PND120. To evaluate the quality of the vocal repertoire, which is often altered in animal models of ASD [57–62], USVs were divided into nine distinct call categories (Fig. 1C). In line with the exploratory sniffing, WT and *Syn2KO* mice emitted a comparable number and spectrum of USVs at PND30, whereas the vocal repertoire of *Syn2KO* mice at PND120 became much less complex (Fig. 1E–G), without affecting peak frequency or amplitude (Supplementary Figure 1B–D), as previously observed [52].

Since adult *Syn2KO* mice experienced epileptic seizures when manipulated, we asked whether the loss of social skills was due to a direct effect of the epileptic attack. To this aim, we performed the male-female social interaction test at various times (1 h, 1 day, and 3 days) after seizure induction. However, the low levels of interaction and the impaired ability to vocalize in *Syn2KO* mice remained unchanged at all time intervals (Supplementary Figure 2), demonstrating that they are not directly linked to the epileptic attack.

Motor behaviors and anxiety represent behavioral functions able to influence social behaviors. Moreover, male and female *Syn2KO* mice were reported to display sex-related differences in the epileptic phenotype ^55^. Thus, we performed the open field test in adult male and female mice at PND120. In both sexes, the total distance traveled and mean speed were comparable to those of sex-matched WT mice (panels A-C in Supplementary Figures 3 and 4). Interestingly, adult male *Syn2KO* mice spent less time in the center of the open field, showing an anxiety-like phenotype (Supplementary Figure 3D), while this phenotype was virtually absent in age-matched female *Syn2KO* mice (Supplementary Figure 4D). Since social behaviors and vocalizations are also typical features of female-female interaction, we also examined the social context and found that *Syn2KO* female mice interact and vocalize similarly to WT mice (Supplementary Figure 4E–G). Taken together, the behavioral observations indicate that only male *Syn2KO* mice represent a model of regressive ASD, and, for this reason, they have been used in all subsequent studies.

### POA-PAG vocal communication network degenerates during development in *Syn2KO* male mice

The lateral periaqueductal gray (LPAG) contains a subpopulation of neurons (PAG-USV) that send excitatory inputs to the downstream vocal premotor area responsible for USV generation [63]. The activity of PAG-USV is tonically inhibited by GABAergic neurons that are, in turn, controlled by afferent inhibitory inputs from the preoptic area of the hypothalamus (POA) [64]. We first characterized the density of excitatory and inhibitory synapses in the LPAG at both PND30 and PND120 by double immunohistochemistry for pre-/post-synaptic markers for inhibitory (VGAT/Gephyrin) and excitatory (VGLUT1/PSD95) synapses.

**Fig. 2:**
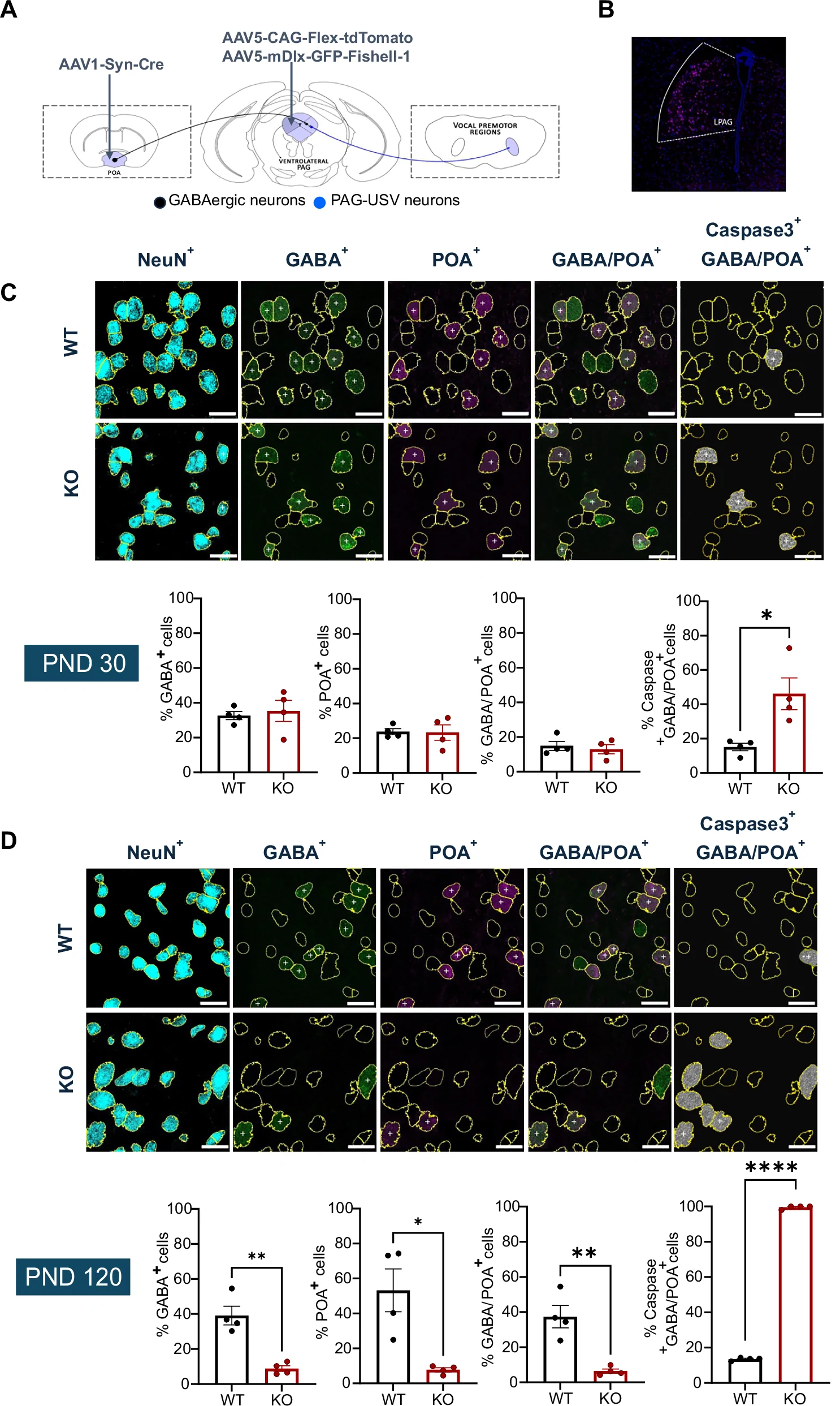
Degeneration of the POA-PAG vocal circuit in adult *Syn2KO* male mice. **(A)** Schematic representation of the transduction strategy used to detect GABAergic neurons in the LPAG area responsible for vocal production. GABAergic afferents from the POA control the activity of these neurons. We transduced the POA area by injecting a transsynaptic AAV vector expressing Cre recombinase (AAV1-hSyn1-Cre) and the LPAG area with AAV vectors expressing a Cre-dependent red fluorescent protein (AAV5-CAG-Flex-tdTomato) to identify neurons in the LPAG that directly receive input from the POA. To specifically select GABAergic neurons that control PAG-USV neurons, the LPAG area was injected with an additional AAV vector GFP under the control of the *Dlx* promoter (AAV5-mDlx-GFP-Fishell-1). **(B)** Representative fluorescence image showing the LPAG region (40x magnification). LPAG was identified using Hoechst staining, and the magenta neurons represent the direct projection from the POA**. (C,D)** *Top:* Representative confocal images of brain slices from WT and *Syn2KO* mice at PND30 (**C**) and PND120 (**D**). PAG slices stained for NeuN^+^ (cyan), GABA^+^ (green), POA^+^ (magenta), GABA/POA^+^ (merge green/magenta), and Caspase-3^+^ (white). Scale bars, 10 µm. White + signs indicate the counted neurons. *Bottom:* Bar plots of the percentages of GABA^+^, POA^+^, GABA/POA^+^ cells with respect to the total number of NeuN^+^ cells and, on the right, the percentage of Caspase-3^+^ cells with respect to the total number of GABA ^+^ cells. *Statistics*. Data are expressed as means ± SEM with superimposed individual experimental points. *p < 0.05; **p < 0.01; ****p < 0.0001 (N = 4 WT and 4 KO mice). **(C)** Unpaired Student’s *t*-test: GABA^+^ analysis, *t*(6) = 0.42, p = 0.69, Cohen’s d = 0.30; POA^+^ analysis, *t*(6) = 0.10, p = 0.92, Cohen’s d = 0.07; GABA/POA^+^ analysis, *t*(6) = 0.53, p = 0.62, Cohen’s d = 0.37; Caspase-3^+^ analysis, *t*(6) = 3.26, p = 0.017, Cohen’s d = 2.31 **(D)** Unpaired Student’s *t*-test: GABA^+^ analysis, *t*(6) = 5.43, p = 0.002, Cohen’s d = 3.84; POA^+^ analysis, *t*(6) = 3.69, p = 0.010, Cohen’s d = 2.61; GABA/POA^+^, *t*(6) = 5.43, p = 0.002, Cohen’s d = 3.84; Caspase-3^+^ analysis, *t*(6) = 130.7, p < 0.0001, Cohen’s d = 92.4.

While no alterations were observed at PND30 in both excitatory and inhibitory synaptic densities, adult *Syn2KO* mice displayed a significant reduction in the density of inhibitory synapses (Supplementary Figure 5), which was also associated with a decrease in the excitatory synapses (Supplementary Figure 6). Based on these observations, we asked whether the marked reduction in the GABAergic input reflected a dysregulation of the POA-PAG inhibitory connections. To identify neurons in the LPAG that directly receive input from the POA, we transduced (i) the POA area by injecting a trans-synaptic adeno-associated virus (AAV) expressing Cre recombinase (AAV1-hSyn1-Cre), and (ii) the LPAG area with AAV vectors expressing a Cre-dependent red fluorescent protein (AAV5-CAG-Flex-tdTomato). Moreover, to specifically select GABAergic neurons that control PAG-USV neurons, the LPAG area was injected with a further AAV vector expressing green fluorescent protein (GFP) under the control of the *Dlx* promoter (AAV5-mDlx-GFP-Fishell-1) (Fig. 2A, B). Using this strategy, we could separately count GABAergic neurons (GABA^+^), neurons receiving afferents from the POA (POA^+^), and GABAergic neurons receiving afferents from the POA (GABA/POA^+^) in the LPAG of young and adult WT and *Syn2KO* mice (Fig. 2C, D). Vocalizing young *Syn2KO* mice showed percentages of neurons in each subpopulation comparable to those of age-matched WT mice (Fig. 2C). On the contrary, non-vocalizing adult *Syn2KO* mice exhibited a strong reduction in the percentage of GABA^+^, POA^+^, and GABA/POA^+^ neurons consistent with the synaptic density data in the same area (Fig. 2D; Supplementary Figure 5). These results indicate dysregulation of the POA-PAG circuit at later developmental stages in *Syn2KO* mice, suggesting that the LPAG is involved in the vocal phenotype of *Syn2KO* mice.

To elucidate the mechanism(s) responsible for the reduction in the GABAergic system in adult *Syn2KO* mice, we stained the LPAG for activated Caspase-3, a marker of apoptosis. In the adult phase, *Syn2KO* mice displayed a marked increase in activated Caspase-3, accounting for the degeneration of GABA/POA^+^ neurons (Fig. 2D, *right panels*) and of the other neuronal subpopulations analyzed (GABA^+^ and POA^+^ neurons; Supplementary Figure 7). Interestingly, a significant, albeit less pronounced, Caspase-3 activation in GABAergic POA-PAG neurons and the other neuronal populations analyzed was already observed in young, normally vocalizing *Syn2KO* mice (Fig. 2C, *right panels*; Supplementary Figure 7). These data highlight a latent period of synaptic and circuit dysregulation in *Syn2KO* mice at PND30, culminating in the erosion of the vocal circuit and the near-total absence of vocal communication at PND120.

### Sensory breakdown parallels the ASD regressive-like phenotype in *Syn2KO* male mice

**Fig. 3:**
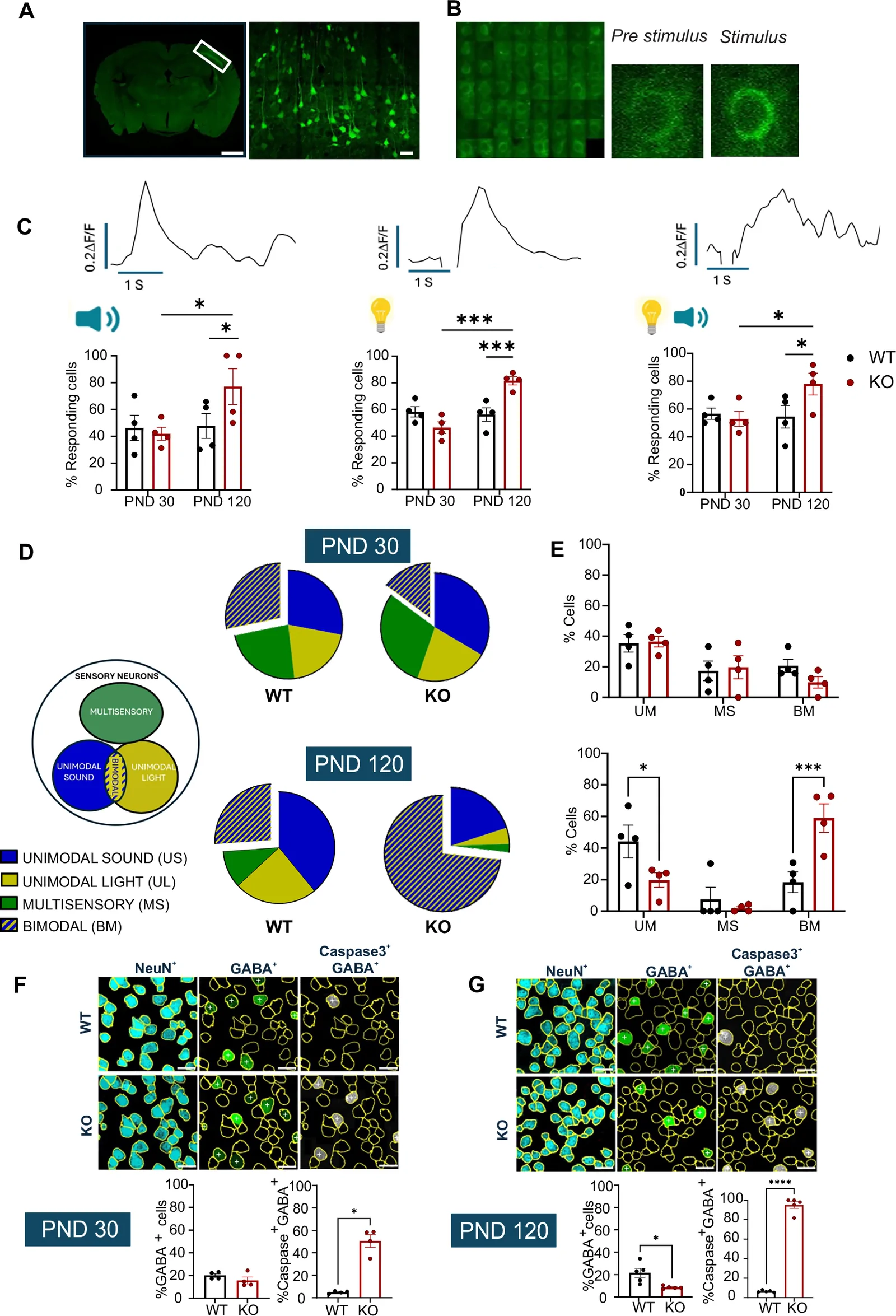
Sensory integration breaks down in PPC of adult *Syn2KO* male mice. **(A)** Representative confocal images of neurons transduced with AAV5-hSyn-jGCaMP8s. *Left:* A coronal brain slice showing GFP expression at the level of PPC in the transduced hemisphere (white rectangle; scale bar, 100 µm). *Right:* Higher magnification of transduced neurons (scale bar, 20 µm). **(B)** *Left:* mosaic of segmented neuronal ROIs acquired using 2-photon calcium imaging. *Right:* a single representative neuron is shown before and after stimulus presentation, revealing the stimulus-evoked increase in fluorescence. **(C)** *Top*: Representative traces of single cellular responses to different stimulations: sound, light, or light and sound. *Bottom:* Percentage of neurons responding to sound, light, or light and sound in young (PND30) and adult (PND120) WT and *Syn2KO* mice. The percentages of responding neurons were calculated as the number of neurons in each category relative to the total number of responding neurons. **(D)** Pie charts display, for each genotype, the percentage of responding cells defined as: *unimodal neurons* (cells responding only to either sound or light; blue and yellow, respectively); *bimodal neurons* (cells responding to both sound and light, delivered individually; dashed yellow), and *multisensory neurons* (cells responding exclusively when sound+light are delivered together; green). The inset visually describes the relationships between the four neuronal subpopulations. **(E)** Bar graphs represent the mean percentages of the four neuronal subpopulations in which the percentage of unimodal neurons (UM) represents the sum of unimodal sound-stimulated and unimodal light-stimulated neurons. **(F,G)** *Top:* Representative confocal images of brain slices from WT and *Syn2KO* mice at PND30 (**F**) and PND120 (**G**). PPC slices were stained for NeuN^+^ (cyan), GABA^+^ (green), and Caspase-3^+^ (white). White + signs indicate the counted neurons. Scale bars, 10 µm. *Bottom:* Bar plots represent the percentages of GABA^+^ neurons with respect to the total number of NeuN^+^ cells, and of Caspase-3^+^ neurons with respect to the total number of GABA^+^ cells. *Statistics*. Data are expressed as means ± SEM with superimposed individual experimental points. *p < 0.05; ***p < 0.001; ****p < 0.0001. **(C)** Mixed-model RM-ANOVA (N = 4 WT PND 30, 96 neurons; 4 KO PND 30, 80 neurons; 4 WT PND 120, 121 neurons; 4 KO PND 120, 152 neurons). Sound protocol, time x genotype interaction: F_1,12_ = 6.65, p = 0.01, ηp² = 0.36 - Fisher’s LSD test: KO-PND30 *vs* KO-PND120, p = 0.0248; WT-PND120 *vs* KO-PND120, p = 0.05; Light protocol, time x genotype interaction F_1,4_ = 157, p = 0.0002, ηp² = 0.98 - Fisher’s LSD test: KO-PND30 *vs* KO-PND120, p = 0.0001; WT-PND120 *vs* KO-PND120, p = 0.0002; Sound+Light protocol, time x genotype interaction F(1,12) = 4.319 p = 0.05, ηp² = 0.27 - Fisher’s LSD test: KO-PND30 *vs* KO-PND120, p = 0.019; WT-PND120 *vs* KO-PND120, p = 0.026. **(E)** Two-way ANOVA. Neuronal subpopulations in young, cell type x genotype interaction: F_2,18_ = 0.92, p = 0.417, ηp² = 0.09; Neuronal subpopulations in adults, cell type x genotype interaction: F_2,18_ = 10.80, p = 0.0008, ηp² = 0.55 - Tukey’s test: WT *vs* KO unimodal neurons, p = 0.0280; bimodal neurons, p = 0.0009). **(F)** Unpaired Student’s *t*-test (N = 4 WT and 4 KO mice): GABA^+^ analysis *t*(6) = 1.284, p = 0.246, Cohen’s d = 0.91; Caspase-3^+^ analysis, *t*(6) = 8.17 p = 0.0286 Cohen’s d = 5.77. **(G)** Unpaired Student’s *t*-test (N = 5 WT and 5 KO mice): GABA^+^ analysis, *t*(8) = 3.23, p = 0.012, Cohen’s d = 2.28; Caspase-3^+^ analysis, *t*(8) = 25.67, p < 0.0001, Cohen’s d ≈ 18.

One of the primary brain regions involved in the integration of sensory stimuli in mice is the PPC [65]. Using 2-photon Ca^2+^ imaging, we recorded the PPC responses to sound (white noise 20–20,000 Hz for 100 ms), light (flashes at 300 lux for 100 ms), and combined light+sound stimuli (Fig. 3A, B). While the responses of young *Syn2KO* mice to all stimuli were similar to those of WT animals, adult *Syn2KO* mice exhibited significantly stronger responses to the various stimulation modalities (Fig. 3C). Thus, the integrative abilities of PPC become dysfunctional after 1 month of age in *Syn2KO* mice.

To dissect which class of neurons was mainly dysregulated, we classified *unimodal neurons* that responded exclusively to either light or sound, *bimodal neurons* that responded to both light and sound, and *multisensory neurons* that responded to light+sound only when delivered simultaneously. Adult *Syn2KO* mice exhibited a decreased response of unimodal neurons, that was mirrored by an increased response of bimodal neurons, while multisensory neurons were comparable to WT mice (Fig. 3D, E). This suggests that the shift from unimodal to bimodal responses, potentially attributable to the GABAergic dysfunction, causes a decline in multimodal sensitivity across development in *Syn2KO* mice. Thus, we evaluated the number of GABA^+^ neurons and their positivity for activated Caspase-3 in the PPC of young (Fig. 3F) and adult (Fig. 3G) WT and *Syn2KO* mice. Similarly to what was observed in the LPAG, young *Syn2KO* mice showed a percentage of GABA^+^ neurons comparable to that of age-matched WT mice, whereas in adult *Syn2KO* mice, GABA^+^ neurons were significantly reduced. Strikingly, already in young *Syn2KO* mice ≈ 50% of inhibitory neurons expressed activated Caspase-3, a percentage that increased to ≈ 90% in adult *Syn2KO* mice. We also extended the analysis to evaluate the density of inhibitory (Supplementary Figure 8) and excitatory (Supplementary Figure 9) synapses in the PPC. As observed in the LPAG, the density of GABAergic synapses, and to a lesser extent of glutamatergic synapses, was significantly reduced in adult *Syn2KO* mice with respect to age-matched WT mice, while the density of both inhibitory and excitatory synapses in young *Syn2KO* mice was indistinguishable from that of WT animals, notwithstanding the observed increase in the activated Caspase-3 signal in GABA^+^ neurons (see Fig. 3F). These data suggest that a general and progressive erosion of the GABAergic system is responsible for the breakdown of sensory integration that parallels the social regression in our model.

### Early and chronic restoration of the inhibitory tonic current rescues the ASD-like phenotype in *Syn2KO* male mice

*Syn2KO* mice experience a defective tonic GABA current responsible for the state of hyperexcitability leading to the epileptic phenotype [50]. THIP, a selective agonist of extrasynaptic GABA_A_Rs, reduces hyperexcitability and ameliorates the epileptic phenotype in the adult, but it is unable to reverse the ASD-related social deficits at this stage of life [51, 52]. Here, we addressed the possibility that sustained treatment with THIP from birth drives proper brain development and rescues social deficits by mimicking the effects of the physiological levels of environmental GABA.

**Fig. 4:**
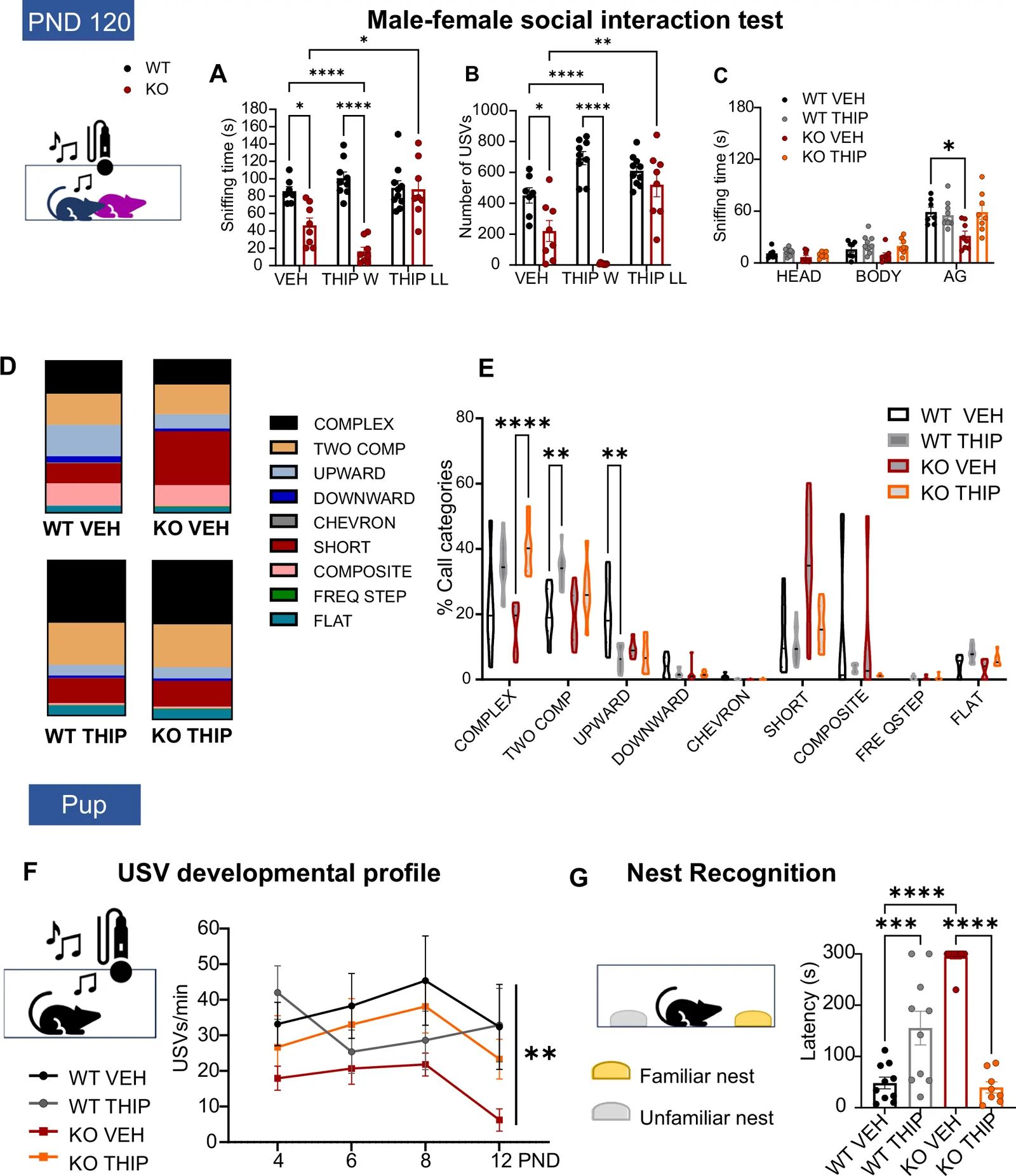
Lifelong treatment with THIP restores social deficits in *Syn2KO* male mice. **(A,B)** Male-female social interaction test performed at PND120 in three experimental groups vehicle-treated mice (Veh; water); THIP W, mice treated with THIP until weaning; THIP LL, mice treated with THIP from birth to adulthood. Bar graphs indicate the time spent in sniffing behaviour expressed as cumulative time (**A**) and total number of USVs emitted during the 3-min interaction (**B**). **(C)** Time spent in social exploration of specific body regions (Head, Body, AnoGenital) by the THIP LL-treated groups (henceforth THIP). **(D)** Colored columns represent the percentage of different syllables emitted by the THIP cohort and calculated as the number of syllables in each category over the total number of calls analyzed in each subject (for details, see Fig. 1C). **(E)** Violin plot depicts the percentage of different syllables emitted by the chronically treated THIP cohorts and calculated as described in D. **(F)** Maternal separation test. Number of USVs emitted by vehicle- and THIP-treated WT and KO pups at PND 4, 6, 8, and 12 during 3-min of maternal separation. **(G)** Nest recognition test performed at PND10 by vehicle- and THIP-treated WT and *Syn2KO* pups. Latency to reach the familiar nest bedding located on one side of a cleaned cage, while the other side was covered with bedding from an unfamiliar nest. *Statistics.* Data are expressed as means ± SEM with superimposed individual experimental points (A-C,F,G). *p < 0.05; **p < 0.01; ***p < 0.001; ****p < 0.0001. **(A)** Two-way ANOVA (N = 7 WT-VEH, 9 WT-THIP W, 10 WT-THIP LL, 8 KO-VEH, 8 KO-THIP W, 8 KO-THIP LL). Genotype effect: F_1,44_ = 39.90, p < 0.0001, ηp² = 0.48; Treatment effect: F_2, 44_ = 7.94, p = 0.0011, ηp² = 0.27; Genotype x Treatment interaction: F_2, 44_ = 13.81, p < 0.0001, ηp² = 0.39 - Tukey’s multiple comparison test: WT-VEH *vs* KO-VEH, p = 0.031; WT-VEH *vs* KO-THIP W, p < 0.0001; WT-THIP W *vs* KO-THIP W, p < 0.0001; KO-VEH *vs* KO-THIP LL, p = 0.0013. **(B)** Two-way ANOVA (N = 7 WT-VEH, 9WT-THIP W, 10 WT-THIP LL, 8 KO-VEH, 8 KO-THIP W, 8 KO-THIP LL). Genotype effect F_1,44_ = 66.73, p < 0.0001, ηp² = 0.60; Treatment effect: F_2, 44_ = 13.59, p < 0.0001, ηp² = 0.38; Genotype x Treatment interaction: F_2,44_ = 19.79, p < 0.0001, ηp² = 0.47 - Tukey’s multiple comparison test: WT-VEH *vs* KO-VEH, p = 0.038; WT-VEH *vs* KO-THIP W, p < 0.0001; WT-THIP W *vs* KO-THIP W, p < 0.0001; KO-VEH *vs* KO-THIP LL, p = 0.0019. **(C)** RM-ANOVA (N = 7 WT-VEH; 10 WT-THIP; 8 KO-VEH, 8 KO-THIP). Body area effect: F_1.449,42.01_ = 163.4, p < 0.0001, ηp² = 0.85; Experimental group effect: F_3,29_ = 5.35, p = 0.0047, ηp² = 0.38; Body area x Experimental group interaction: F_6, 58_ = 3.01, p = 0.0126, ηp² = 0.24 - Tukey’s multiple comparison test: WT-VEH *vs* KO-VEH p = 0.0160 (AG). **(E)** Mixed-model ANOVA: experimental group x call category interaction: F_24,261_ = 6.67, p < 0.0001, ηp² = 0.38 - Tukey’s multiple comparison test: Complex KO-VEH *vs* KO-THIP, p < 0.0001; two-component WT-VEH *vs* WT-THIP, p = 0.0147; upward WT-VEH *vs* WT-THIP, p = 0.0414. **(F)** RM-ANOVA (N = 10 WT-VEH; 10 WT-THIP; 14 KO-VEH, 9 KO-THIP). Treatment effect: F_3,41_ = 4.92, p = 0.0052, ηp² = 0.17. **(G)** One-way ANOVA (N = 10 WT-VEH; 10 WT-THIP; 14 KO-VEH, 9 KO-THIP). F_3, 38_ = 51.94, p < 0.0001, η² = 0.80 - Tukey’s multiple comparison test: WT-VEH *vs* WT-THIP, p = 0.0007; WT-VEH *vs* KO-VEH, p < 0.0001; KO-VEH *vs* KO-THIP, p < 0.0001).

We preliminarily assessed whether THIP is transferred to the pups via maternal lactation. To this aim, we analyzed the plasma of the pups at PND15 using a targeted liquid chromatography-tandem mass spectrometry (LC-MS/MS). The compound was clearly detected in the plasma of THIP-treated mothers’ pups, indicating that pups were indeed exposed to the drug from birth (Supplementary Figure 10). Next, we tested two time windows for the THIP treatment: a chronic treatment throughout the entire lifespan from birth to adulthood (THIP LL) or a shorter treatment from birth to weaning (THIP W). We first evaluated the drug efficacy in restoring sociability in adult *Syn2KO* mice using the male-female social interaction test. We observed that only THIP LL, but not THIP W, rescued the sniffing levels, particularly in the AG area, and the number of USVs (Fig. 4A–C). Based on this observation, only the chronic THIP treatment was performed in the subsequent studies. The vocal repertoire of THIP-treated *Syn2KO* mice showed increased complexity, with significantly increased emission of complex and two-component calls, fully approaching the vocal repertoire of WT animals (Fig. 4D, E). We also checked the effects of the chronic THIP treatment in the open field test, in which adult *Syn2KO* male mice display anxiety and stereotyped behaviors (see Supplementary Figure 3 and ^51^). THIP increased the time spent in the center and reduced the duration of repetitive grooming behaviors, indicating a positive effect of chronic THIP treatment on various behavioral aspects (Supplementary Figure 11).

Because *Syn2KO* pups exhibit early developmental alterations during the first two postnatal weeks, we tested pup vocalizations during the maternal separation test. At this stage, THIP treatment of *Syn2KO* mice effectively reversed the drastic decline in vocalizations by increasing the number of USVs emitted at PND8 and PND12 (Fig. 4F). To better characterize the maternal attachment in pups, we also performed a nest recognition task, in which animals had to discriminate between their home bedding and unfamiliar bedding placed in different corners of a new cage. *Syn2KO* pups did not reach the corner with their home bedding during the 5-min test, suggesting that the social deficit was not restricted to USV emissions (Fig. 4G). Also, in this task, THIP effectively reduced the latency to reach the correct corner, thereby restoring maternal attachment behavior. It is worth noting that the same treatment negatively affected WT pup performance by altering the USV profile in the maternal separation test and increasing the nest recognition latency (Fig. 4F, G).

Given the co-morbidity between epileptic and social phenotypes in *Syn2KO* mice, we also monitored the seizure onset in THIP-treated and untreated mice. Unexpectedly, all mice experienced epileptic seizures, and the Kaplan-Meier analysis of seizure occurrence shows that the treatment did not affect the time of onset of overt epilepsy (Supplementary Figure 12A, B). The lack of effect of chronic THIP on the epileptic propensity of *Syn2KO* mice was confirmed by electrophysiological studies on cortico-hippocampal slices using high-density multielectrode arrays (HD-MEA). *Syn2KO* slices displayed a significantly increased frequency of interictal events with respect to WT slices, but the level of network hyperexcitability was comparable in THIP-treated and untreated *Syn2KO* slices (Supplementary Figure 12C–I). This further confirms that social deficits in our model are not causally linked to the epileptic phenotype and that the early restoration of tonic inhibition during development is disease-modifying for ASD-genesis, but not for epileptogenesis.

### Early restoration of the inhibitory tonic current rescues the POA-PAG circuit dysfunction in *Syn2KO* male mice

**Fig. 5:**
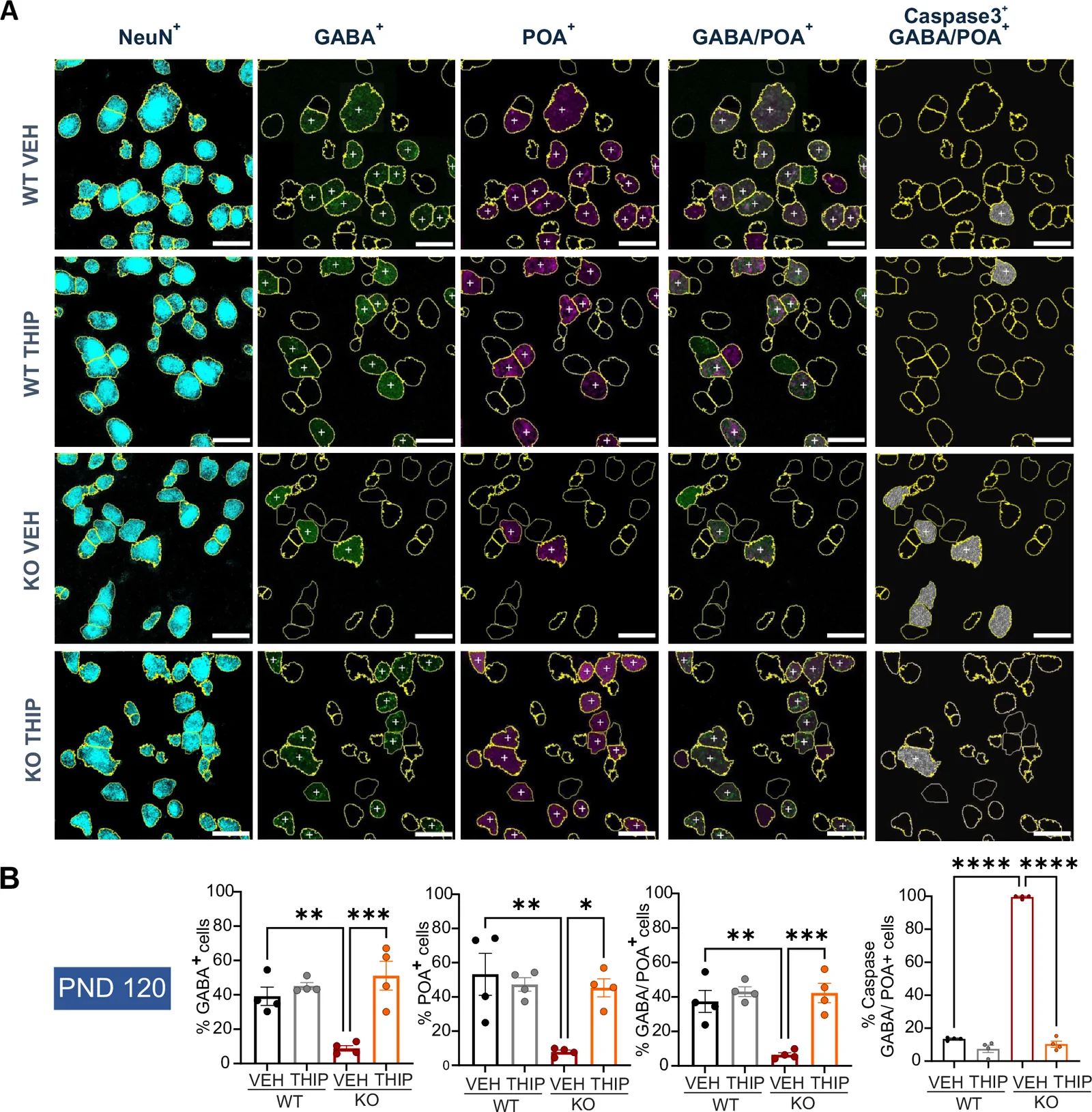
Early restoration of the GABAergic tonic current prevents the degeneration of the POA-PAG circuit in adult *Syn2KO* male mice. **(A)** Representative confocal images of LPAG slices from adult WT and *Syn2KO* mice treated with VEH or THIP from birth to adulthood. Images represent brain slices stained for NeuN^+^ neurons (cyan) that are GABA^+^ (green), POA^+^ (magenta) or GABA/POA^+^ (merge green/magenta), and Caspase-3^+^ (white). White + signs indicate the counted neurons. Scale bars, 10 µm. **(B)** Bar graphs represent the percentages of GABA^+^, POA^+^, GABA/POA^+^, and Caspase-3^+^ in GABA/POA^+^ cells with respect to the total number of NeuN^+^ cells. *Statistics*: Data are expressed as means ± SEM with superimposed individual experimental points. *p < 0.05; **p < 0.01; ***p < 0.001; ****p < 0.0001 (N = 4 WT-VEH, 4 WT-THIP, 4 KO-VEH, 4 KO-THIP). One-way ANOVA: GABA^+^ F_3, 12_ = 13.30, p = 0.0004, η² = 0.77 - Tukey’s multiple comparison test: WT-VEH *vs* KO-VEH, p = 0.0063, KO-VEH *vs* KO-THIP, p = 0.0004; POA^+^ F_3, 12_ = 8.77, p = 0.0024, η² = 0.69 - Tukey’s multiple comparison test: WT-VEH *vs* KO-VEH, p = 0.0030; KO-VEH *vs* KO-THIP, p = 0.0116); GABA/POA^+^ F_3,12_ = 14.80, p = 0.0002, η² = 0.7872 - Tukey’s multiple comparison test: WT-VEH *vs* KO-VEH, p = 0.0020; KO-VEH *vs* KO-THIP, p = 0.0006; Caspase-3^+^ F_3,12_ = 931.6, p < 0.0001, η² = 0.9957 - Tukey’s multiple comparison test: WT-VEH *vs* KO-VEH, p < 0.0001; KO-VEH *vs* KO-THIP, p < 0.0001.

We next evaluated the POA-PAG connections to verify whether the positive effects of chronic THIP treatment observed in *Syn2KO* mice were associated with a recovery of the vocal circuit (Fig. 5A). The analysis of the LPAG revealed that, while THIP had no effect in WT animals, the impaired percentages of GABA^+^, POA^+^, and GABA/POA^+^ neurons in treated adult *Syn2KO* mice were fully rescued by the chronic THIP treatment to the levels of WT animals (Fig. 5B). Additionally, the levels of activated Caspase-3, markedly increased by the *Syn2* deletion in GABA^+^, POA^+^, and GABA/POA^+^ neurons, were strongly decreased in THIP-treated *Syn2KO* mice, reaching the low levels observed in WT mice (Fig. 5B; Supplementary Figure 13). We also assessed synaptic density in the LPAG. We observed a full rescue of inhibitory synaptic density in THIP-treated *Syn2KO* mice that was associated with an increased expression of both presynaptic and postsynaptic inhibitory markers (Supplementary Figure 14). Notably, THIP also increased the expression of the excitatory presynaptic marker VGLUT1 that was depressed in *Syn2KO* mice (Supplementary Figure 15). These findings emphasize the key role of tonic inhibition in shaping the socio-vocal circuit across development and underline the importance of very early correction of the E/I imbalance to rescue the function of the POA-PAG circuit and contrast the regressive phenotype of *Syn2*KO mice.

### THIP treatment alleviates multisensory impairments in adult male *Syn2KO*

**Fig. 6:**
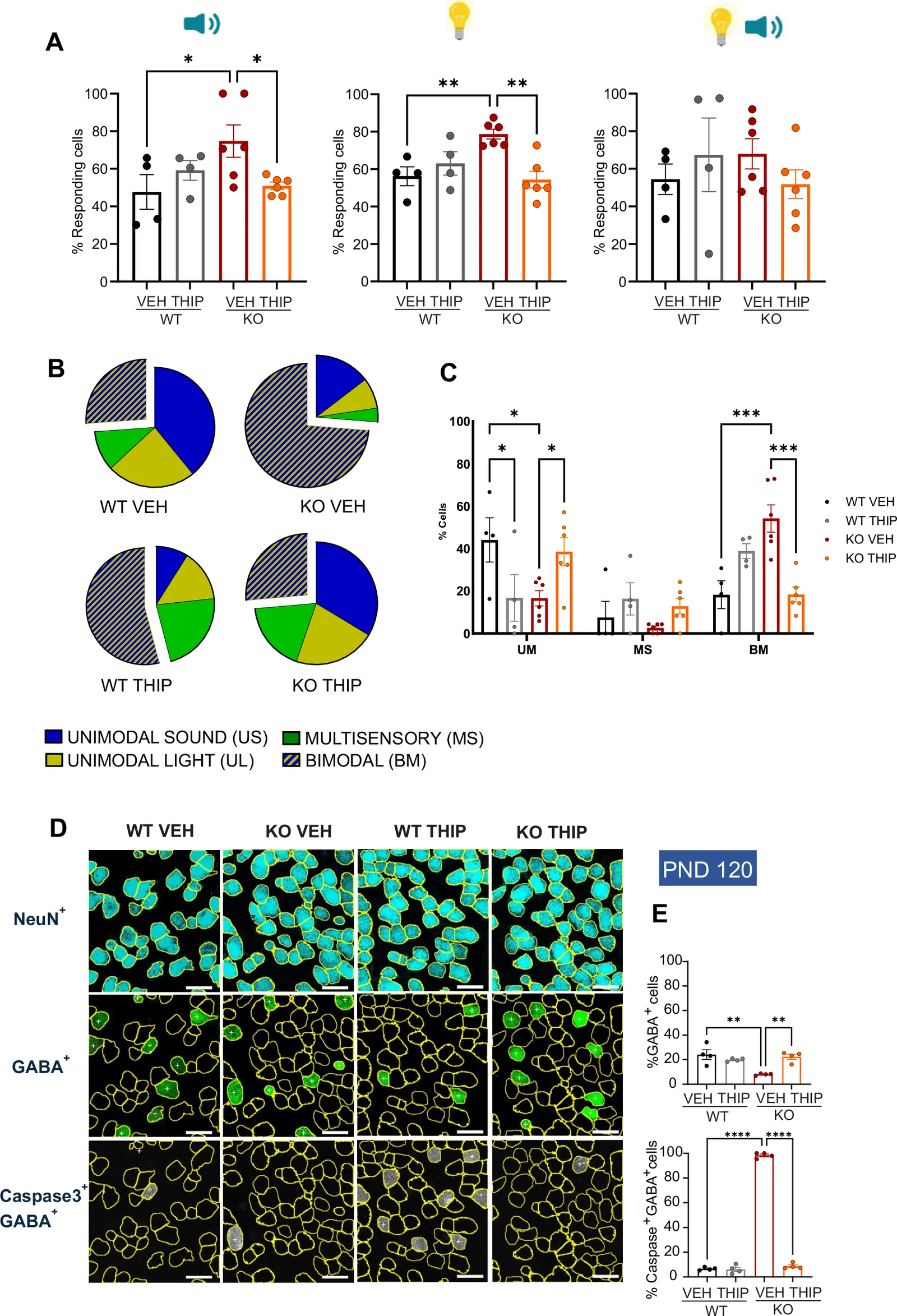
Early restoration of the GABAergic tonic current rescues sensory integration in adult *Syn2KO* male mice. **(A)** Percentage of neurons responding to sound, light, or light + sound in adult WT and *Syn2KO* mice treated with either VEH or THIP from birth to adulthood. The percentages of responding neurons were calculated as the number of neurons in each category relative to the total number of responding neurons. **(B)** Pie charts display, for each genotype and treatment, the percentage of responding cells defined as: *unimodal neurons* (cells responding only to either sound or light; blue and yellow, respectively); *bimodal neurons* (cells responding to both sound and light, delivered individually; dashed yellow), and *multisensory neurons* (cells responding exclusively when sound + light are delivered together; green). **(C)** Bar graphs represent the mean percentages of the four neuronal subpopulations, in which the percentage of unimodal neurons (UM) represents the sum of unimodal sound-stimulated and unimodal light-stimulated neurons. **(D)** Representative confocal images of PPC slices from adult WT and *Syn2KO* mice treated with either VEH or THIP from birth to adulthood. Images represent NeuN ^+^ neurons (cyan) that are GABA^+^ (green), and Caspase-3^+^ (white). White + signs indicate the counted neurons. Scale bars, 10 µm. **(E)** Bar graphs represent the percentages of GABA^+^ neurons with respect to the total number of NeuN^+^ cells, and Caspase-3^+^ neurons with respect to the total number of GABA^+^ neurons. *Statistics.* Data are expressed as means ± SEM with superimposed individual experimental points. *p < 0.05, **p < 0.01, ***p < 0.001. **(A)** One-way ANOVA (N = 4 WT-VEH; 121 neurons; 4 WT-THIP, 126 neurons; 6 KO-VEH, 204 neurons; 6 KO-THIP, 166 neurons). Sound: F_3,16_ = 3.413, p = 0.043, R² = 0.3902 - Bonferroni’s test: WT-VEH *vs* KO-VEH, p = 0.0288; KO-VEH *vs* KO-THIP, p = 0.0315; light: F_3,16_ = 7.110, p = 0.0030, R² = 0.5714 - Bonferroni’s test: WT-VEH *vs* KO-VEH, p = 0.0018; KO-VEH *vs* KO-THIP, p = 0.0088); Light+Sound: F_3,16_ = 0.6663, p = 0.5849, R² = 0.1111. **(B)** Two-way ANOVA. Neuron type x Experimental group interaction: F_6,48_ = 7.99, p < 0.0001, ηp² = 0.50 - Bonferroni’s test: Unimodal cells: WT-VEH *vs* KO-VEH, p = 0.0147; WT-VEH *vs* WT-THIP, p = 0.0331; KO-VEH *vs* KO-THIP, p = 0.0382; Bimodal cells: WT-VEH *vs* KO-VEH, p < 0.0007; KO-VEH *vs* KO-THIP, p = 0.0002. **(C)** One-way ANOVA (N = 4 WT-VEH, 4 WT-THIP, 4 KO-VEH, 4 KO-THIP mice). GABA^+^: F_3,12_ = 10.22, p = 0.0013, η² = 0.72 - Tukey’s test: WT-VEH *vs* KO-VEH, p = 0.0015; KO-VEH *vs* KO-THIP, p = 0.0038. Caspase-3^+^: F_3,12_ = 1400, p < 0.0001, η² = 0.997 - Tukey’s test: WT-VEH *vs* KO-VEH, p < 0.0001; KO-VEH *vs* KO-THIP, p < 0.0001.

To determine whether rescuing social deficits parallels the recovery of sensory integration, we performed 2-photon Ca^2+^ imaging in the PPC of THIP-treated WT and *Syn2KO* mice. While chronic THIP treatment had no detectable effects in WT mice, it specifically and significantly decreased the percentage of neurons responding to sound and light in *Syn2KO* mice, bringing it to levels comparable to those of untreated or treated WT mice (Fig. 6A). A more detailed analysis revealed that chronically THIP-treated *Syn2KO* mice displayed a significant decrease in the percentage of bimodal neurons responding to both sound and light, and a mirror significant increase of unimodal neurons reaching, for both categories, the levels observed in WT mice, thus normalizing the neuronal specialization (Fig. 6B, C). To ascertain whether the restoration of multimodal specificity was also associated with a recovery of the GABAergic system and connectivity, we evaluated the number of GABA^+^ neurons and their positivity for activated Caspase-3 in the PPC of THIP-treated adult WT and *Syn2KO* mice. THIP fully corrected the depopulation of GABA^+^ neurons and, consistently, virtually abolished the increased Caspase-3 activation observed in vehicle-treated adult *Syn2KO* mice (Fig. 6D, E). We also measured synaptic density in the PPC of THIP-treated *Syn2KO* mice and observed that both inhibitory and excitatory contacts reached levels comparable to those of WT animals. As in the PAG, we observed a full recovery of both presynaptic and postsynaptic inhibitory markers (Supplementary Figure 16) and an increase in excitatory contacts driven by an increased expression of the presynaptic marker VGLUT1 (Supplementary Figure 17). Thus, boosting the tonic inhibitory current during development effectively restored WT-like global sensory responses in *Syn2KO* animals.

## Discussion

Deficits in social interactions are supportive of alterations in sensory processing [66, 67]. Accordingly, ASD patients exhibit deficits in sensory and multisensory integration/processing [68–71]. We studied the *Syn2KO* mouse, a well-characterized model of ASD, by showing that its regressive social phenotype occurs after a latent period of deterioration of GABAergic function that involves the brain areas committed to vocalization and complex sensory processing. Accordingly, the sustained enhancement of the GABAergic tonic current by chronic postnatal treatment with THIP was able to rescue the regressive ASD-like phenotype of *Syn2KO* mice.

### *The Syn2KO* mouse is a model of regressive ASD with defects in vocalization and multisensory integration

We demonstrated that the behavioral phenotype of *Syn2KO* mice recapitulates social regression. Notably, mutations in *SYN2*, the human gene encoding the synaptic vesicle phosphoprotein Synapsin II (SynII) are associated with neurodevelopmental disorders, specifically ASD and epilepsy [72, 73]; https://gene.sfari.org/database/human-gene/SYN2; SFARI gene, score 2).

We investigated the developmental timeline of social performances of the *Syn2KO* male mouse by studying young mice at PND30, a critical time window for development and plasticity, and adult mice at PND120. We show that the social abilities of *Syn2KO* mice are intact in young mice, but they decline in adults in terms of both sniffing behavior and USV emission. We previously showed that social deficits and reduced USV emission during maternal separation were present in *Syn2KO* mice early after birth [52]. We extend these findings and define a new picture for the social development of *Syn2KO* male mice in which social abilities recover at PND30, but they undergo a regressive phenotype in adulthood. This represents clear preclinical evidence that the regressive phenotype observed in the *Syn2*KO model can be viewed as a multiphasic process characterized by a mixed pattern of early delays, a latent period of apparently normal development, and a subsequent decline [9, 15].

We characterized the POA-PAG vocal circuit to investigate the mechanisms responsible for the decline in vocal communication observed in adult mutant males. We showed that the degeneration of the vocal circuit begins at a young age but becomes phenotypically overt only at adulthood. This was paralleled by a delayed deterioration of cortical responses to sensory stimuli, with reduced activation of PPC neurons specific to distinct stimulus modalities (unimodal neurons) that was compensated by a marked activation of bimodal neurons not distinguishing the input sensory modalities. Although we do not provide direct evidence for a causal relationship between sensory and social deficits, our findings indicate that both domains are associated with a broader dysregulation of the GABAergic system in males. We also show that the effects of the *Syn2* deletion in males, which are latent during the CP, manifest in early life and in adulthood, resulting in severe social deficits. The same social profile was not detected in *Syn2KO* females, possibly due to sex differences in the modulation of the GABA_A_Rs (see below). Future studies are needed to determine whether females are fully protected from GABAergic degeneration or whether they engage in sex-specific mechanisms able to mask the social deficits, but not the epileptic propensity. As social camouflaging in females is often present as a key feature of neurodevelopmental disorders [74], particularly ASD, our model could also represent a promising platform to explore the mechanisms underlying this aspect of social impairment.

### GABAergic dysfunctions are associated with developmental defects in *Syn2*KO male mice

SynII is an abundant synaptic vesicle phosphoprotein that plays a paramount role in synaptogenesis and synaptic transmission [75–78]. The absence of SynII primarily affects GABAergic transmission by virtually abolishing asynchronous GABA release, which is mainly responsible for activating eGABA_A_Rs. This culminates in the appearance of overt epileptic seizures at 3-4 months of age [50, 51, 53, 79, 80]. GABA is the primary inhibitory neurotransmitter in the brain and plays a crucial role in orchestrating brain development, particularly in modeling connectivity and plasticity [33]. Tampering with GABAergic signaling during early postnatal life delays the onset of the CP and abolishes the activity-dependent synaptic refinement [81, 82]. Conversely, reactivation of GABA_A_Rs using benzodiazepines restores the CP time course [81], demonstrating the crucial function of inhibition in shaping the correct brain development. Extrasynaptic GABA_A_Rs, responding to very low concentrations of environmental GABA, generate the tonic GABA current that provides a constant inhibition essential in regulating neuronal excitability [33]. This critical role is consistent with the increased expression of eGABA_A_Rs in the mouse brain during development [83]. During this period, the eGABA_A_R drive has a fundamental role in regulating circuit formation and excitability. The functional importance of tonic inhibition is maintained in the adult brain, where eGABA_A_Rs participate in the homeostatic responses to neuronal hyperactivity [84]. However, the precise time windows in which these two functions are active and their potential overlap during the postnatal life remain to be clarified. In our study, young *Syn2KO* male mice started to be affected by a latent impairment of the GABAergic system in both LPAG and PPC despite their still intact social repertoire and sensory integration. PAG represents a major integrative hub receiving inputs from multiple brain regions. However, in the present study, we specifically focused on the POA–PAG circuit because of its well-established role in the generation of USVs, which were profoundly impaired in our model^65-66^. The PPC is a key brain region involved in multisensory integration and plays an important role in the interpretation of socially relevant cues. We show that both brain areas undergo a latent GABAergic dysfunction during the developmental window, which may contribute to the emergence of the regressive phenotype observed in adulthood, when we detected a widespread degeneration of the GABAergic system. While our findings identify strong associations between POA–PAG circuit alterations and vocal deficits, a limitation of the study is that we were unable to directly establish causality through optogenetic manipulation because *Syn2*KO mice exhibited a marked fragility following invasive procedures. To date, we do not know the causes of neuronal loss, but several mechanisms may be responsible, including glutamate excitotoxicity, inflammation, and oxidative/metabolic stress, as suggested from studies on neurodegenerative diseases [85–88]. Since our observations reveal Caspase-3 activation in young mice, when GABA tonic current is already altered [89], but seizures and social deficits are not established, we hypothesize that neuronal death may result from a cumulative sub-threshold excitotoxicity.

The absence of social deficits in *Syn2KO* females can be attributed to sex-specific compensatory effects on the impaired GABAergic transmission. We previously showed that a low glycemic index diet aimed at boosting tonic inhibition, restrains epileptogenesis in a sex-specific fashion [53]. The antiepileptic effect of diet on seizures in females was due to an increase in cortical allopregnanolone, a neurosteroid present in high levels in females during diestrus and known to enhance GABAergic transmission. In this context, we cannot exclude that the appearance of the social phenotype in females is prevented by the compensatory effects of neurosteroids on the low endogenous stimulation of eGABA_A_Rs.

### Sustained reinstatement of the tonic GABA current is a disease-modifying treatment for the regressive ASD-like phenotype

We previously demonstrated that one-week chronic treatment of adult *Syn2KO* mice with the eGABA_A_R agonist THIP, a drug currently on trial for Fragile X and Angelman syndromes [90, 91], efficiently counteracted the occurrence of epileptic seizures [51]. However, the treatment did not modify the severe ASD-like traits of adult male mutant mice [52], suggesting that the social phenotype depends on anomalous high-order connectivity resulting from altered network homeostasis during development. Interestingly, we show here that sustained enhancement of the inhibitory tonic current by THIP from birth to adulthood markedly rescues the phenotype in vocalization and social interactions, ensures the physiological development and integrity of the POA-PAG circuit, and preserves the physiological mechanisms of sensory specificity and multimodal integration in PPC cortical neurons. This appears to be a developmental effect, as the same treatment fails to suppress the epileptic phenotype in adulthood, which most likely depends on an E/I imbalance in the medial temporal lobe. However, the positive effects of the treatment are not maintained when THIP is discontinued at weaning, suggesting that, at least in our model, stimulation of eGABA_A_Rs is required not only during the CP but also thereafter to maintain behavioral circuits, a function distinct from its role in regulating network excitability.

Several hypotheses can be advanced to explain the basis of the dissociation between social and epileptic phenotypes. One possibility is that tonic inhibition plays a dual role in shaping circuit formation during development and in regulating neuronal excitability in the mature brain. This suggests the existence of two non-overlapping temporal windows for these effects, although they might still influence each other. An alternative explanation is that chronic activation of eGABA_A_Rs by THIP leads to progressive receptor desensitization, increasing neuronal excitability over time, while their role in circuit development during CP persists [92]. Another important aspect concerns the role of phasic inhibitory currents that may be indirectly affected by our treatment. Phasic inhibition, mainly mediated by fast spiking parvalbumin interneurons, controls network synchrony and epileptic discharges. In contrast to tonic inhibition, the maturation of phasic interneurons is independent of circulating GABA levels and is stimulated by their activity-dependent recruitment during development [93]. Thus, early chronic stimulation of eGABA_A_Rs during development may be irrelevant for the maturation of fast-spiking interneurons, leaving epileptogenesis unaffected. Finally, we must consider that the developmental changes in GABA_A_R structure are age- and sex-dependent, region-specific, and can be gradual or transient across postnatal development, adulthood, and aging [94, 95].To elucidate the mechanisms by which long-term THIP treatment exerts differential effects on behavioral profiles and neuronal excitability in our model, it will be crucial to characterize the changes in receptor subunit composition occurring during development and dissect the specific effects of THIP on their functional properties. Our data in *Syn2KO* mice suggest that GABA-dependent wiring and GABA-dependent modulation of excitability act simultaneously, with weights that change across developmental time windows.

## Conclusions

Social regression in *Syn2*KO male mice involves the emergence of behavioral deficits following a latent developmental period associated with synaptic alterations. Despite transient social deficits in early postnatal life, *Syn2*KO mice develop an overt ASD-like phenotype in adulthood, after a latent period of GABA dysfunctions that affect neuronal specificity, sensory integration, and brain connectivity. Our findings suggest that social regression in this ASD model follows a multiphasic process characterized by a mixed pattern of early delays within a silent ASD-genesis period of typical development, followed by a progressive decline. The rescue of the regressive phenotype upon THIP-mediated enhancement of tonic GABAergic signaling during the CP indicates the fundamental role of inhibition in shaping brain connectivity. THIP was previously investigated in clinical studies for Fragile X syndrome and showed promising preclinical effects in Rett syndrome models [90, 96]. Among neurodevelopmental disorders, Rett and Fragile X syndromes, two of the most extensively studied conditions for investigating developmental regression, are monogenic syndromes in which preclinical studies identified alterations in the GABA tonic current^37,38,40,43^. Thus, our observations on behavioral regression are consistent with evidence from other genetic models, supporting the possibility that latent GABAergic dysfunction may represent a shared feature across at least a subset of monogenic neurodevelopmental syndromes associated with developmental regression.

## Materials and methods

### Animals

*Syn2KO* mice were backcrossed to a C57BL/6 J background (Charles River, Calco, Italy) for more than 10 generations. HET male X HET female mice were mated to obtain WT and KO experimental animals. Mice were housed in groups under a standard 12-hour light/dark cycle with *ad libitum* access to a standard diet and water. All experiments were performed with the experimenters blinded to genotype and treatment. All procedures involving animals and their care were carried out in accordance with the guidelines established by the European Commission (Directive 2014/26/EU) and were approved by the Italian Ministry of Health (Authorizations #226-2024-PR and #245-2025-PR).

### THIP administration

In pharmacological experiments, THIP (MedChem; Cat. No.: HY-10232) treatment was chronically administered from the day of birth. The effects of the treatment were evaluated across two temporal windows. In one cohort, the treatment was administered from birth until weaning (THIP W), whereas in the second cohort it was administered throughout the animals’ lifespan (THIP LL). THIP (200 mg/L) was delivered to the mother dissolved in drinking water starting from PND0 (day of birth) to PND21 (weaning day) for transferring to the offspring via lactation. Water was used as a vehicle. After weaning, THIP (20 mg/L) or vehicle was administered directly to the pups via drinking water. Doses were chosen based on Zhong et al. [41], and the post-weaning dose was comparable to those reported by Medhrian et al. 2015 and Michetti 2017. Mice were subjected to the following battery of behavioral tests: maternal separation test (PND 4, 6, 8, 12); nest recognition (PND10); seizure detection starting from PND30; social interaction PND30 and PND120. At the end of behavioral observations, a subgroup of mice was used for ex vivo HD-MEA recordings. Three separate batches of mice were subjected to viral injections around PND90 for the evaluation of in vivo 2-photon Ca^2+^ imaging in PPC, analysis of POA-PAG circuits and GABAergic neurons in PPC at PND120.

### Behavioral experiments

#### Seizure detection

After weaning, we performed a weekly cage change to monitor seizure onset. This novelty stimulus triggers a seizure in our mouse model [80]. Days of latency for the first seizure were calculated, and once the first attack appeared, the social interaction test was performed. The evaluation of social behavior was repeated 1 h, 1 day, and 3 days after the seizure appearance to evaluate the effect of brain hyperexcitability on social performance.

#### Open field

To assess locomotion and anxiety, adult mice underwent an open field test. Each mouse was placed in an opaque open-field box (40x40x40 cm) and recorded with an Anymaze camera (Stoelting, Wood Dale, IL) mounted 20 cm above the apparatus. The test started by individually placing each animal in the center of the arena and then recording its spontaneous exploration of the apparatus for 10 min. Between each test session, the floor of the apparatus was cleaned with 50% ethanol. Total distance, and time spent in the center and on the border, were automatically collected and analyzed using the Anymaze camera and software, respectively.

#### Social interaction test

In the male-female social interaction test, male mice were tested for 3-min at PND30 and PND120 as in Michetti et al. [52]. The male mouse was isolated before the test (1 h at PND30; from 1 h to 3 days at PND120). The vaginal estrous condition of each female stimulus was assessed before the interaction to select only females in estrous for the test. The social interaction was scored for the duration of the following behavioral responses performed by the subject mouse: AG (direct contact with the anogenital area), body sniffing (sniffing or snout contact with the flank area), nose-to-nose sniffing (sniffing or snout contact with the head/neck/mouth area). The cumulative sniffing time across different body areas was also calculated. For the female-female interaction, we analyzed the same parameters and used the same protocol, with test females isolated for 1 h before the test. In both social contexts, USVs were recorded during the social interaction through an ultrasonic microphone (Avisoft UltraSoundGate condenser microphone capsule CM16; Avisoft Bioacoustics, Berlin, Germany) mounted 10 cm above. The microphone was sensitive to frequencies between 10 and 180 kHz. Vocalizations were recorded using the Avisoft recorder software (version 3.2). Settings included sampling rate at 250 kHz: format 16 bit. For acoustic analysis, recordings were transferred to Avisoft SasLab Pro (version 4.40). The following parameters were analyzed: number of calls, mean call duration, and qualitative and quantitative analyses of sound occurrence, measured by frequency and amplitude at the spectrum peak. The syllable repertoire was analyzed only for the male-female interaction following the previously described categorization [57]: *Complex*, one sound component containing two or more directional changes in frequency with varying direction and rate of changes; *Two-component*, composed by a main call with an additional short component of higher frequency; *Upward*, one element with a continuous increase in frequency (at least 6.25 kHz more than the frequency at the beginning of the vocalization); *Downward*, one component with a constant decrease (at least 6.25 kHz less than the initial frequency of the vocalization); *Chevron*, ‘inverted-U shape’ with a constant increase followed by a decline; *Short*, one component shorter than 5 ms; *Composite*, two parallel lines consisting of a constant sound; *Frequency steps*, three components composed by a main call resembling the composite calls flanked by two discontinuous “step”; *Flat*, one component at constant frequency. The percentage of different calls emitted was calculated as the number of syllables in each category/total number of calls.

#### Maternal separation

USVs emitted by pups in response to a 3-min of maternal separation test were evaluated at PND4, 6, 8, and 12 as in Michetti et al. [52]. On the testing day, each pup was placed into an empty plastic container (5 × 10 cm), located inside a sound-attenuating polystyrene box. USVs were recorded with an ultrasonic microphone (Avisoft UltraSoundGate condenser microphone capsule CM16, Avisoft Bioacoustics, Berlin, Germany). The microphone was placed on the top of the polystyrene box, about 10 cm above the plastic container. Vocalizations were recorded and counted as described above.

#### Nest recognition test

Each pup was separated from the dam at PND10 and placed at the center of a standard plexiglass cage containing familiar bedding on one side, clean bedding at the canter, and bedding coming from an unknown litter on the other side. The latency to reach the familiar bedding was analyzed with a cut-off set at 5 min.

### Stereotaxic injections for transduction

For 2-photon Ca^2+^ imaging experiments, mice were injected with AAV encoding the Ca^2+^ indicator jGCaMP8s (∼1 × 1013 vg/m; pGP-AAV-syn- jGCaMP8s-WPRE; Addgene #162374) in PPC (AP: -2.2 mm, ML: 2.5 mm; DV: 0,3 mm). For the characterization of the POA-PAG circuit, animals were injected in: (i) POA (AP = 0.14 mm, ML = 0.3 mm, DV = 5.5 mm) with an anterograde trans-synaptic AAV expressing Cre recombinase (AAV1-hSyn1-Cre; Addgene # 105553); (ii) LPAG area (AP = − 4.7 mm, ML = 0.7 mm, DV = 1.75 mm), with AAVs expressing a Cre-dependent red fluorescent protein (AAV5-CAG-Flex-tdTomato, Addgene #28306,) and GFP under the control of the *Dlx* promoter (AAV5-mDlx-GFP-Fishell-1, Addgene #83900). This strategy allowed the identification of neurons in the LPAG that directly receive input from the POA and specifically select GABAergic ones. For all injections, mice were placed in a stereotaxic frame with their heads fixed to a flat skull position. A small craniotomy was performed bilaterally at the injection coordinates indicated above, and AAVs were injected via a glass pipette (0.1 μL in POA with AAV1-hSyn1-Cre; 1 μL in LPAG with AAV5-CAG-Flex-tdTomato and AAV5-mDlx-GFP-Fishell-1 (1:1), at a flow rate of 0.1 μL/min). The total volume of viral preparation was slowly injected into the target area over a 2-min period for POA and 10 min for LPAG. To prevent backflow of the virus, the electrode was held in place for 5 min before slowly retracting. After the injection, mice were returned to their home cage and administered with Diclofenac (1 mg/kg) and Baytril (1 mg/kg) to reduce inflammation and speed up recovery from anesthesia. After brain surgery, animals were single-housed with standard environmental enrichment (nest material and plastic house) and *ad libitum* access to food and water, and allowed to recover for at least 4 weeks.

### In-vivo 2-photon calcium imaging

Mice were anesthetized, and a cranial window (5-mm diameter) was created using a dental drill. After removing the skull in the area of interest, the cover glass (5-mm diameter) was glued with dental cement (Kulzer, Paladur). In addition, a customized metal headplate was glued with dental cement on the anterior portion of the cover glass to allow a stable fixation of the animal’s head during the acquisition session. Mice were administered Diclofenac (2.5 mg/kg) and Baytril (1 mg/kg) to reduce inflammation. Four hours later, the imaging acquisition session began, and animals were subjected to three stimulation protocols: (i) flashlight (300 lux for 100 ms); (ii) sound (white noise with a range of 20–20,000 Hz for 100 ms); (iii) light+sound stimulation. The speaker for sound generation was positioned ∼6 cm from the animal and triggered by the Avisoft program (Avisoft-SasLab Pro version 4.40). The light source was positioned ∼10 cm from the mouse. An acoustic-optic deflector (AOD) 2-photon microscope (Femtonics, FEMTO 3D ATLAS) with a water immersion objective (Nikon, N16XLWD-PF 16x) was used. The field of view was selected based on the blood vessels map acquired during the cranial window surgery. Each animal was acquired for 10 sessions of 5 min each. A region of interest (ROI) was manually drawn for each neuron at various depths, and the Ca^2+^ signals of each ROI were acquired. For somatic Ca^2+^ imaging, images were acquired at 500 × 500 pixels at 30 kHz, with a typical field of view of 300 × 300 µm. All data were analyzed offline by MESc software and MATLAB 2016b (MathWorks, USA). The Ca^2+^ signal of each neuron was calculated as the relative fluorescence change Δ F/F = (F-F_0_)/F_0_ over time. For each ROI, the F_0_ value was calculated as the average signal recorded 1 s before stimulation. Detection of Ca^2+^ transients was performed by thresholding the peak amplitude and rising rate. We defined the noise level as 3-fold the standard deviation (SD) of the baseline activity. To classify the neuronal subpopulations, we defined (i) *unimodal neurons*, i.e., neurons responding exclusively to light or to sound; (ii) bimodal *neurons*, i.e., neurons responding to both light and sound delivered separately; (iii) *multisensory neurons*, cells responding only to the association of light+sound. The percentage of responding neurons in each category was calculated as the number of neurons in a subpopulation with respect to the total number of responding neurons.

### Labeling of POA-PAG connections

LPAG contains a subpopulation of neurons (PAG-USV) responsible for producing USVs and whose activity is controlled by afferent inhibitory inputs from the POA [63, 64]. We labeled the specific POA-PAG connections by injecting the POA with a transsynaptic AAV vector expressing Cre recombinase (AAV1-hSyn1-Cre) and the LPAG area with AAV vectors expressing a Cre-dependent red fluorescent protein (AAV5-CAG-Flex-tdTomato). To allow the identification of the GABAergic neurons in the LPAG, this area was transduced with a further AAV vector expressing GFP under the control of the Dlx promoter (AAV5-mDlx-GFP-Fishell-1). Young (PND30) and adult (PND120) male mice were sacrificed 3 weeks after the stereotaxic injections, and their brains were processed as detailed below. Thirty μm-thick tissue sections were imaged with a 63× immersion oil objective with a Leica SP8 confocal microscope (Leica Microsystem).

### Immunohistochemistry and image analysis

The mice were deeply anesthetized with an intraperitoneal injection of ketamine/xylazine and transcardially perfused with ice-cold 0.1 M phosphate buffer (PB; pH 7.4), followed by 4% paraformaldehyde (PFA) in 0.1 PB. After perfusion, brains were dissected and post-fixed in PFA solution overnight at 4 °C. After 3 washes in 0.1 M PB, brains were cryoprotected by immersion in 30% sucrose solutions and cut in 30-μm sections with a vibratome. Bain slices were stored at −20 °C in a solution containing 30% ethylene glycol and 20% glycerol in 0.1 M PB. PAG sections were processed for free-floating immunofluorescence. After a blocking step in phosphate-buffered saline (PBS) containing 0.05% Triton X-100 and 10% normal goat serum (NGS), sections were incubated overnight at 4 °C with primary antibodies, washed, and incubated for 2 h at 25 °C with secondary antibodies (anti-rabbit/anti guineapig Alexa Fluor 488, and anti-mouse Alexa Fluor 647). For the study of excitatory and inhibitory synapses in the LPAG and PPC, we used the following primary antibodies diluted in blocking solution: rabbit anti-vesicular GABA transporter (vGAT; 1:1000, Synaptic Systems 131002), guinea pig anti-VGLUT-1 (1:250, Synaptic Systems 135304), mouse anti-postsynaptic density 95 (PSD95, 1:1000, Thermo Fisher Scientific, MA1-045), and mouse anti-gephyrin (1:1000, Synaptic Systems 147011). Double immunofluorescence (vGAT/Gephyrin or vGLUT1/PSD95) was performed with the simultaneous addition of the primary antibodies. Sections were washed in PBS (4 × 10 min) and incubated with secondary antibodies for 2 h. After several rinses with PBS, sections were mounted on glass slides and acquired with a Leica SP8 confocal microscope (Leica Microsystem) at 63x. The fluorescence intensity and the co-localization of synaptic markers were analyzed as previously described [97, 98]. We acquired three images from each slice, and three ROIs were analyzed for each image. Confocal microscope settings were kept the same for all experiments. For the quantitative analysis of vGAT and vGLUT1 Z-series stacks of 15 consecutive confocal sections (1024 × 1024 pixels) spaced by 2 μm were acquired at 63X. Images from both channels were overlaid, and background labeling was subtracted. Both vGLUT1/PSD95- and vGAT/Gephyrin-positive colocalizations were calculated from the Manders coefficients (M1) output by the JACoP plugin in the Fiji distribution of ImageJ software.

For the study of POA-PAG connections, the LPAG sections were immunostained with the following primary antibodies: NeuN (1:250, Synaptic Systems 266004) and Caspase-3 (1:100, RD system AF835). To isolate the subtypes of LPAG neurons (ROIs for GABA^+^, POA^+^, and GABA/POA^+^ neurons), the NeuN channel Z-max projection was binarized and filtered for shapes > 10 μm. The same protocol was used to study GABA degeneration in PPC. Then, the ImageJ Analyze Particles plugin was used to outline the different neuronal subpopulations. The ROIs were then used to measure the raw fluorescence intensity of GABA^+^, POA^+^, and GABA/POA^+^ neurons. Three control ROIs were used to calculate the average background fluorescence intensity to consider GABA, POA, GABA/POA positively expressed. Data are shown as the number of positive cells in each subpopulation divided by the total number of NeuN^+^ positive cells. Subsequently, the same procedure was performed on different slices to evaluate Caspase-3^-^positive cells. Data related to Caspase-3 are represented by the number of Caspase-3^+^ cells divided by the total number of GABA^+^, POA^+^, and GABA/POA^+^, respectively.

### LC-MS/MS

Plasma samples were centrifuged at 21,100 x g for 20 min at 4 °C. An aliquot of plasma was extracted (1:3) with cold CH_3_CN containing 200 nM of metformin as internal standard. Calibration curves were prepared in blank mouse plasma over a 10 nM to 100 µM range. Three quality control samples were prepared by spiking the parent compound into blank mouse plasma in final concentrations of 0.2, 2, and 20 µM. The calibrators and quality control samples were extracted (1:3) with the same extraction solution as the plasma samples. The plasma samples, calibrators, and quality control were centrifuged at 3,270 x g for 20 min at 4 °C. The supernatants were analyzed by LC-MS/MS on a SCIEX 7500+ system equipped with an OptiFlow® Pro ion source and connected to an Integrated ExionLC AE system consisting of a Pump, Autosampler, Column Oven, Degassing unit, and System Controller from AB SCIEX Instruments. Electrospray ionization was applied in positive mode. Compound-dependent parameters, as MRM transitions and collision energy were developed for the parent compound and the internal standard. The analyses were run on a Restek Raptor POLAR X column (100 × 2.1 mmID, particle size 2.7 µm) with an ACE Excel UHPLC Pre-column filter at 35 °C, using H_2_O + 0.1% HCOOH (A) and MeCN + 0.1% HCOOH (B) as mobile phase. All samples (plasma calibrators and quality controls) were quantified by MRM peak area response factor to determine the levels of the parent compound in the plasma.

### HD-MEA recordings

Treated and untreated *Syn2KO* mice were subjected to electrophysiological recordings between PND120 and PND150 to evaluate the effects of THIP treatment on hippocampal network excitability.

#### Slice preparation

Mice were deeply anesthetized by inhalation of isoflurane and sacrificed. Brains were quickly removed from the skull, blocked, and immersed in an ice-cold solution, and containing (in mM): 252 sucrose, 26 NaHCO_3_, 25 KCl, 20 CaCl_2_, 10 MgSO_4_, 12.5 KH_2_PO_4_, and 10 glucose [99] that had been continuously oxygenated (95% O_2_-5%CO_2_, 4 °C). Next, 300-µm horizontal slices were cut on a Leica VT1200S vibratome in the same ice-cold solution. Slices were transferred for 60 min to a recovery chamber filled with a pre-warmed and continuously oxygenated (95% O_2_-5%CO_2_, 34 °C) artificial cerebrospinal fluid solution (ACSF) composed (in mM): 125 NaCl, 2.5 KCl, 2 CaCl_2_, 1 MgSO_4_, 1.25 NaH_2_PO_4_, 26 NaHCO_3_, and 25 glucose [99]. Last, slices were kept in the same ACSF at room temperature (RT, 22 °C) for 30 min before HD-MEA recordings.

#### Recordings and analysis

Epileptiform activity was recorded using the MaxOne system (MaxWell Biosystems, Zurich, Switzerland) in acute cortico-hippocampal slices over multiple (n = 2–5) 3-min sessions following a 30-min stabilization period. Spontaneous local field potentials (LFPs), in the form of interictal (I-IC) events, were induced by applying 400 µM 4-aminopyridine (4-AP). Signals were acquired at 20 kHz and subsequently lowpass filtered (cutoff: 200 Hz). I-IC events were identified as waveforms exceeding 5.5 standard deviations from the baseline signal. Following the initial detection of events across all electrodes, a filtering process was applied to ensure reliable I-IC event identification. Multi-electrode events were detected by analyzing time histograms of event occurrences across all electrodes using 200-ms bins. Events exceeding 1.5 times the standard deviation of the histogram distribution were considered valid. Additionally, multielectrode events were excluded if they involved fewer than 10 electrodes. For detailed event analysis, the observation window was centered on each detected event. Multi-electrode I-IC events were further analyzed by binning the data in 5-ms intervals around each event to examine their temporal distribution. To eliminate artifacts, a synchrony-based filtering approach was applied: if the peak (distribution-center bin) of the temporal distribution exceeded 50% of the total distribution count, the event was considered excessively synchronous and thus classified as an artifact. Valid interictal event features, including frequency, instantaneous frequency, and bursting properties, were analyzed to characterize event patterns. All signal preprocessing, interictal event detection, and post-processing filtering were performed using custom MATLAB scripts (The MathWorks, R2024a).

### Statistical analysis

The number of samples required for the experiments was preliminarily calculated based on experimental variability and the need to achieve an appropriate number of replications for robust statistical analysis. The number of animals was predetermined using the G*Power software according to the following formula: *n*  =  Z^2^ × σ^2^ /Δ^2^, where Z is the value of the distribution function f(α,β) (with α and β type-I and type-II errors, respectively; based on α  =  0.05 and 1-β  =  0.9), σ is the standard deviation of the groups (set between 0.2-0.3 based on similar experiments and preliminary data) and Δ the minimum percent difference that is thought to be biologically relevant (0.2 or 20%). Experiments were carried out blindly to the experimenter. Data are expressed as means ± SEM for the number of independent animals (n) and shown with the superimposition of the individual experimental points. Normal distribution was assessed using the D’Agostino–Pearson or Shapiro normality test. The unpaired or paired two-tailed Student’s *t*-test was used to compare two normally distributed sample groups. To compare two sample groups that were not normally distributed, the Mann-Whitney’s *U*-test was used. To compare more than two normally distributed sample groups, One-way, two-way, or Mixed-Model ANOVA followed by post-hoc multiple comparisons (Bonferroni’s or Tukey’s tests). To compare more than two non-normally distributed sample groups, we used the Kruskal-Wallis one-way ANOVA followed by the post-hoc Dunn’s test. p < 0.05 was considered significant. To compare seizure latency, Kaplan–Meier survival analysis was used. Statistical analysis was carried out using Prism v9 (GraphPad Software).

## Supplementary information

Supplementary Figures

## Data Availability

All data supporting the findings of this study are available within the article and its supplementary files. Any additional requests are available from the corresponding author on reasonable request.
