# Supplementary material for: Social regression stems from a latent phase of GABAergic dysfunction and sensory breakdown in a model of synaptopathy
Source: Mol Psychiatry. 2026 Jul 13;31(12):6905–21. doi: 10.1038/s41380-026-03751-9 (PMC13736689; doi:10.1038/s41380-026-03751-9)
Supplement: Supplementary file 1 [file 41380_2026_3751_MOESM1_ESM.pdf]

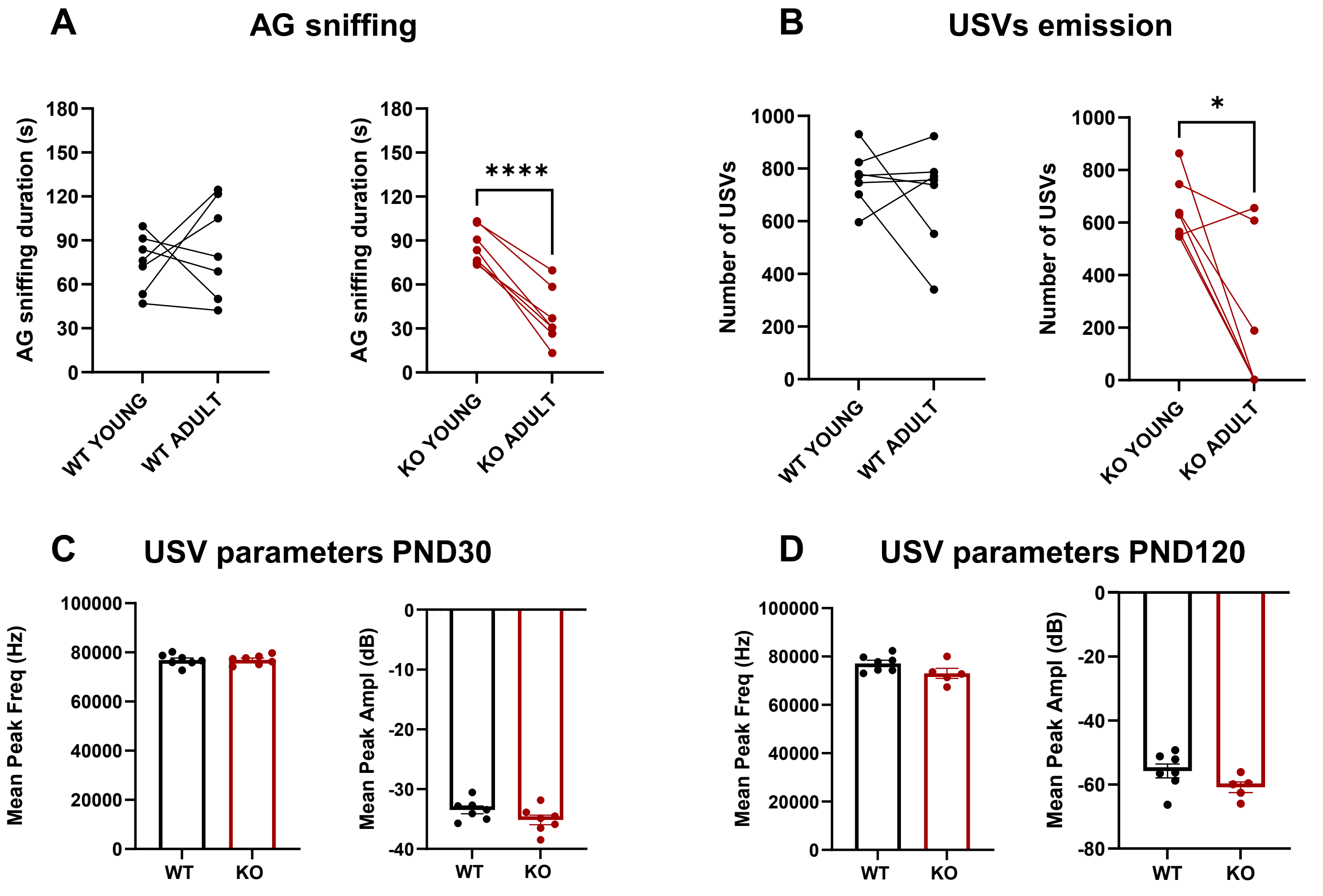

Supplementary Figure 1. Evolution of social and vocal parameters during development in WT and *Syn2KO* mice. (A,B) Duration of social sniffing in the anogenital (AG) area (**A**) and USV emission (**B**) evaluated in each subject of WT and KO mice longitudinally tested in a male-female social interaction test at PND30 and PND120. **(C,D)** Mean peak frequency and amplitude of USVs emitted by young (PND30, **C**) and adult (PND120, **D**) WT and KO mice during the male-female social interaction test.

Statistics. Data are expressed as means ± SEM with superimposed individual experimental points. *p<0.05; ****p<0.0001 (N = 7 WT and 7 KO). **(A)** Paired Student's *t*-test: WT, *t*(6) = 0.62, p=0.56, Cohen's dz = 0.23; KO, *t*(6) = 9.79, p<0.0001, Cohen's dz = 3.70; **(B)** Paired Student's *t*-test: WT, *t*(6) = 0.84, p=0.43, Cohen's dz = 0.32; KO, *t*(6) = 3.60, p=0.011, Cohen's dz = 1.36. **(C)** Unpaired Student's *t*-test: mean peak frequency *t*(12) = 0.11, p=0.92, Cohen's d = 0.06; mean peak amplitude (12) = 1.63, p=0.13, Cohen's d = 0.94. **(D)** Unpaired Student's *t*-test: mean peak frequency *t*(10) = 1.80, p=0.10, Cohen's d = 1.14; mean peak amplitude *t*(10) = 1.72, p=0.12, Cohen's d = 1.09.

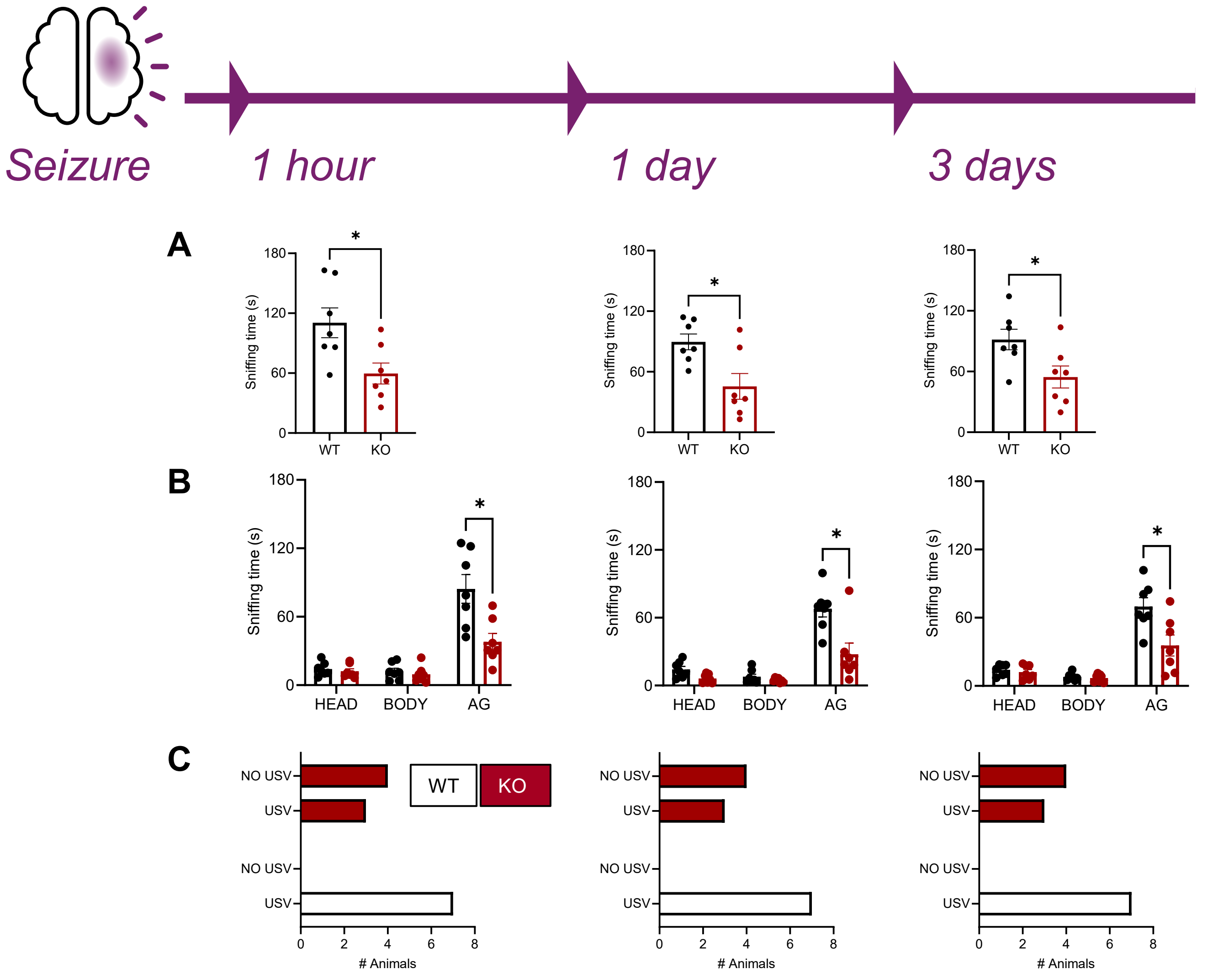

Supplementary Figure 2. Social deficits in adult *Syn2KO* male mice are not caused by seizure activity. (A,B) Cumulative time of social exploration (**A**) and sniffing duration in each body area (Head, Body, AG; **B**) during a male-female social interaction test repeated 1 hour, 1 day, and 3 days after the seizure. **(C)** Bar graphs represent the number of vocalizing *vs* non-vocalizing WT and KO mice recorded during the interaction with a receptive female.

Statistics. Data are expressed as means ± SEM with superimposed individual experimental points (A,B). *$p<0.05$ (N = 7 WT and 7 KO). **(A)** Unpaired Student's *t*-test. 1-h post: $t(12) = 2.79$, p=0.017, Cohen's d = 1.61; 1-day post: $t(12) = 2.95$, p=0.012, Cohen's d = 1.71; 3-day post: $t(12) = 2.50$, p=0.028, Cohen's d = 1.44. **(B)** Two-way ANOVA. 1-h post: genotype x body area interaction $F_{2,24} = 10.23$, p=0.0006, $\eta p^2=0.4$ - Bonferroni's test: AG, WT *vs* KO, p=0.0308. 1-day post: genotype x body area interaction $F_{2,24} = 7.84$, p=0.0024, $\eta p^2=0.40$ - Bonferroni's test. AG: WT *vs* KO, p=0.0215. 3-day post: genotype x body area interaction $F_{2,24} = 8.40$, p=0.0017, $\eta p^2=0.41$ - Bonferroni's test: AG, WT *vs* KO, p=0.0470.

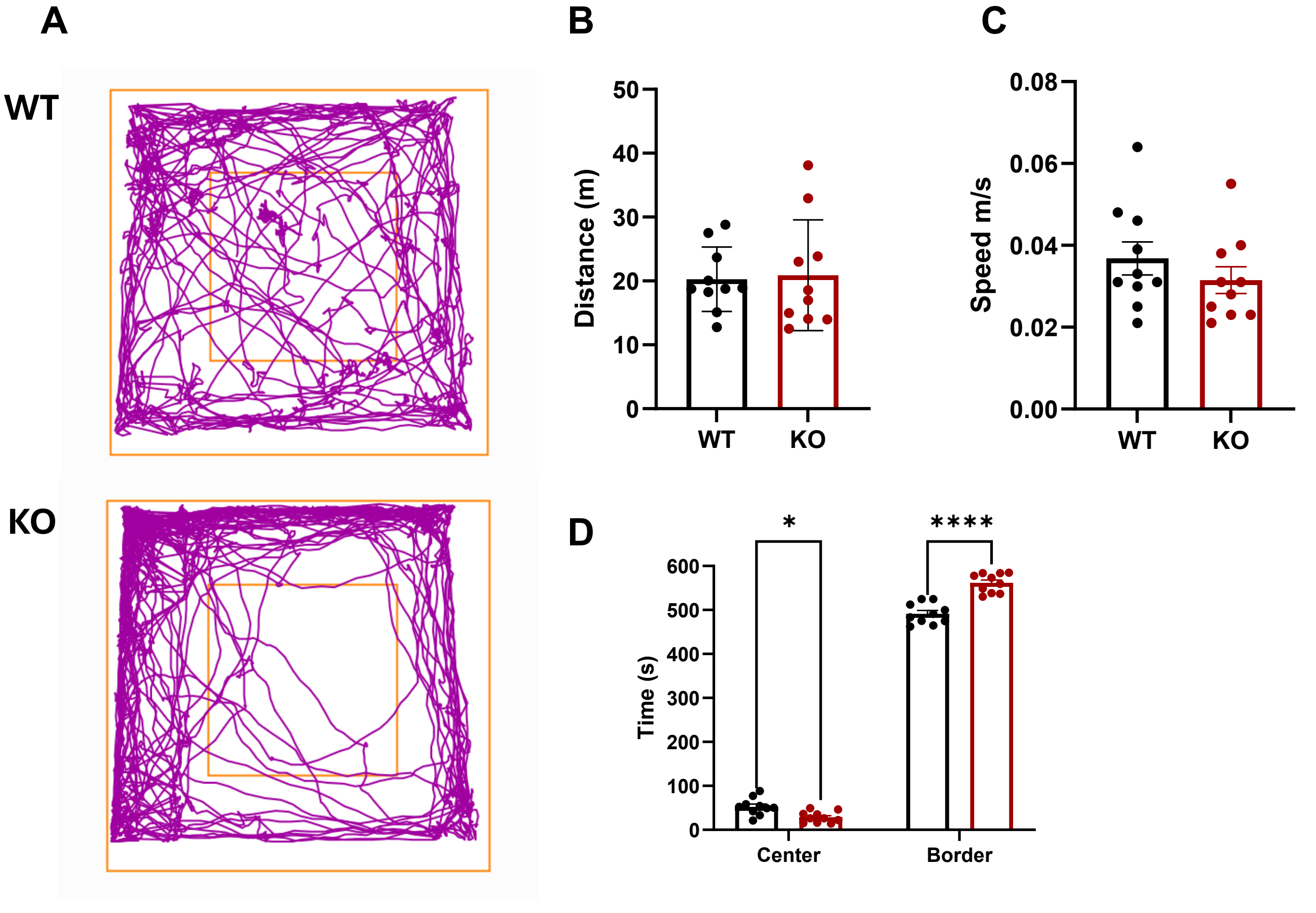

Supplementary Figure 3. Increased anxiety in adult *Syn2KO* male mice. (A) Representative tracking of locomotor activity performed by WT and KO mice during a 10-min open field test. **(B-D)** Distance travelled, mean speed, and time spent in the borders versus the center of the arena.

Statistics. Data are expressed as means ± SEM with superimposed individual experimental points. *p<0.05; ****p<0.0001 (N = 10 WT and 10 KO). **(B,C)** Unpaired Student's *t*-test. Distance: *t*(18) = 0.20, p=0.85, Cohen's d = 0.09; Speed: *t*(18) = 1.02, p=0.32, Cohen's d = 0.48. **(D)** Two-way ANOVA. Genotype x Zone interaction: $F_{1,36}$ = 57.30, p<0.0001, ηp^2=0.61 - Bonferroni's test: center WT *vs* KO, p=0.0202; border WT *vs* KO, p=0.0308.

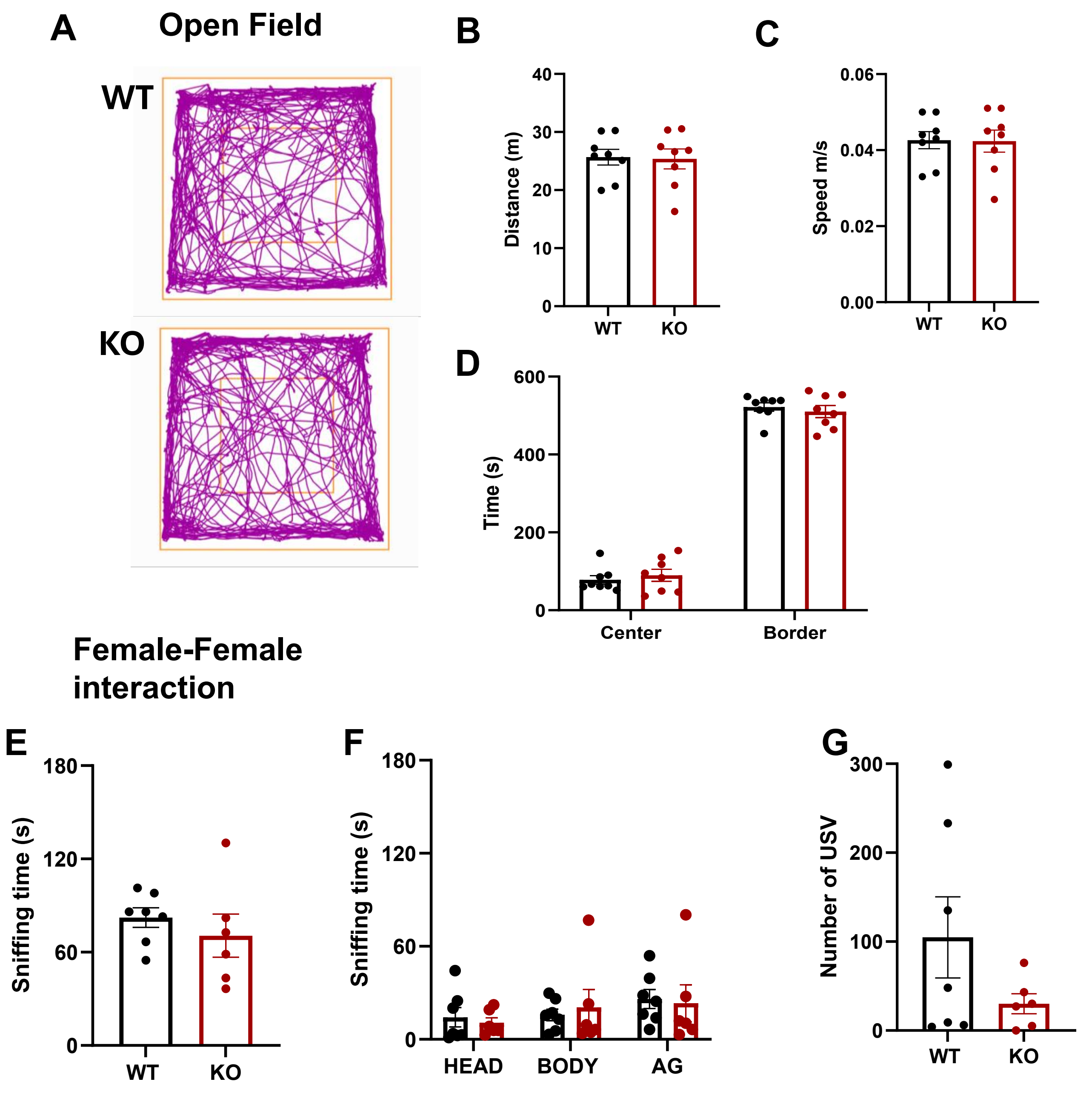

Supplementary Figure 4. Adult Syn2KO female mice do not show behavioral deficits. (A) Representative tracking of locomotor activity performed by WT and KO female mice during a 10-min open field test. **(B-D)** Distance travelled, mean speed, and time spent in the borders versus the center of the arena. **(E-F)** Cumulative time of social exploration (**E**) and sniffing duration in each body area (Head, Body, AG, **F**) during a female-female social interaction test. **(G)** Number of USVs emitted during the female-female social encounter.

Statistics. Data are expressed as means ± SEM with superimposed individual experimental points (N = 7 WT and 7 KO). **(B,C,E,G)** Unpaired Student's *t*-test. Distance: $t(14) = 0.14$, p=0.89, Cohen's d = 0.08; Speed: $t(14) = 0.068$, p=0.95, Cohen's d = 0.04; Sniffing: $t(14) = 0.14$, p=0.89, Cohen's d = 0.08; USVs: $t(11) = 1.47$, p=0.17, Cohen's d = 0.89; **(D,F)** Two-way ANOVA. Time, genotype x zone $F_{1,28} = 0.779$, p=0.385, $\eta p^2=0.027$; Sniffing, genotype x body area $F_{2,33} = 0.187$, p=0.831, $\eta p^2=0.011$.

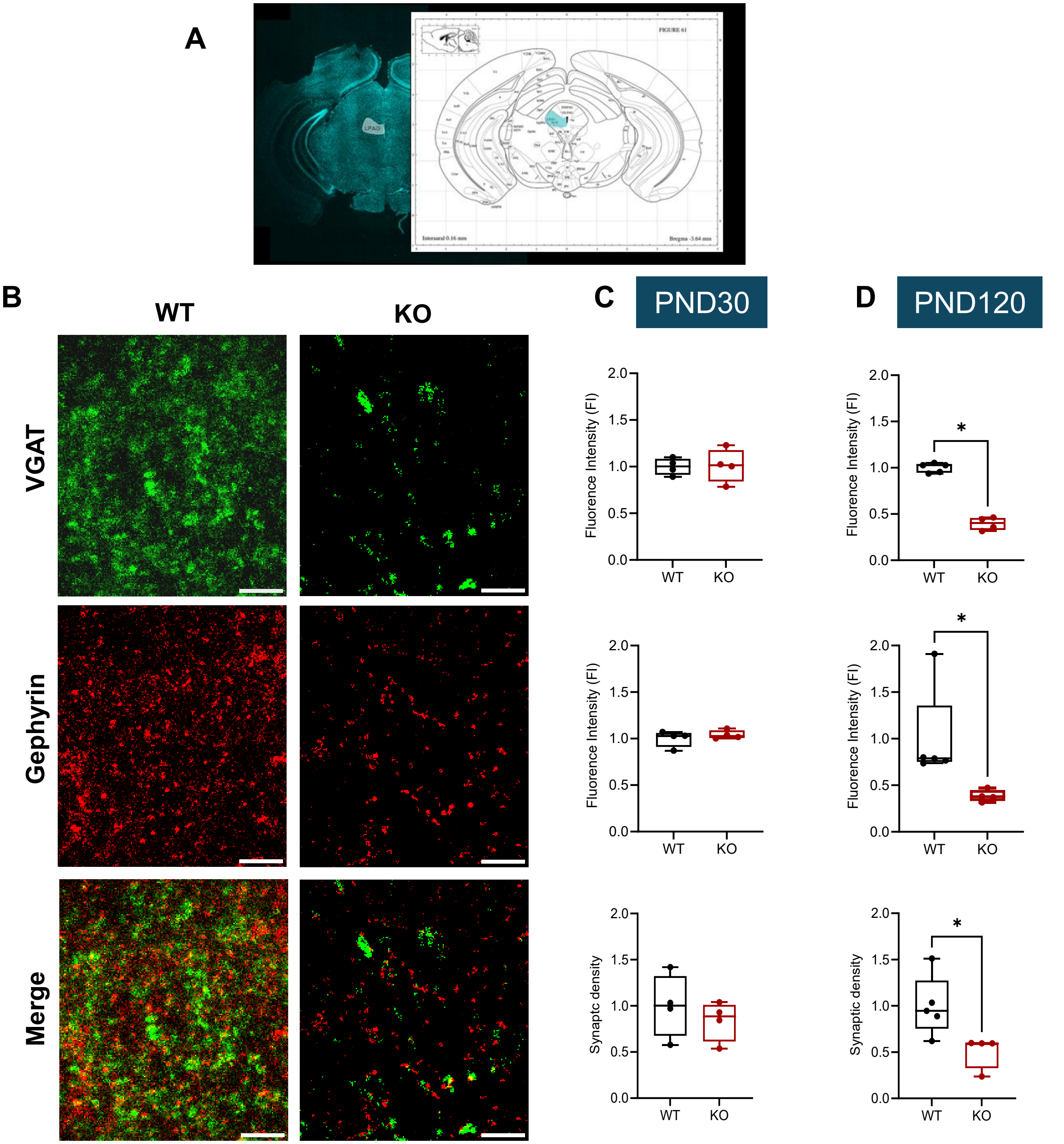

Supplementary Figure 5. Reduction of inhibitory synapses in the LPAG parallels the loss of vocalization ability in adult *Syn2KO* male mice. (A) *Left:* Representative coronal brain section showing a low magnification of the LPAG area (white). *Right:* Section from *the* Paxinos' atlas showing the anatomical location of the LPAG (labelled in blue). (**B**) Representative confocal images of LPAG from adult WT and *Syn2KO* mice acquired using a 63x oil immersion objective. Brain slices were immunostained for vGAT and Gephyrin. The bottom row displays the colocalization of the two signals used to identify synaptic contacts. Scale bars, 10 µm. (**C,D**) Box plots show the quantification of fluorescence intensity for vGAT, Gephyrin, and the density of inhibitory synapses (#synapses/μm^2). Analyses were conducted on slices from young (PND30; **C**) and adult (PND120; **D**) mice.

Statistics. Box plots are characterized by median (center line), 25th and 75th percentiles (box), and min-max values (whiskers). $*p<0.05$ (N = 4 WT and 4 KO for PND30; N = 5 WT and 4 KO for PND120). (**C**) Mann-Whitney's *U*-test. vGAT: $U = 8$, $p>0.999$; Gephyrin: $U = 7$, $p>0.8857$; Synaptic density: $U = 5$, $p=0.4857$. (**D**) Mann-Whitney's *U*-test. vGAT: $U = 0$, $p=0.015$; Gephyrin: $U = 0$, $p=0.016$, $r \approx 0.67$; Synaptic density: $U = 0$, $p=0.0195$.

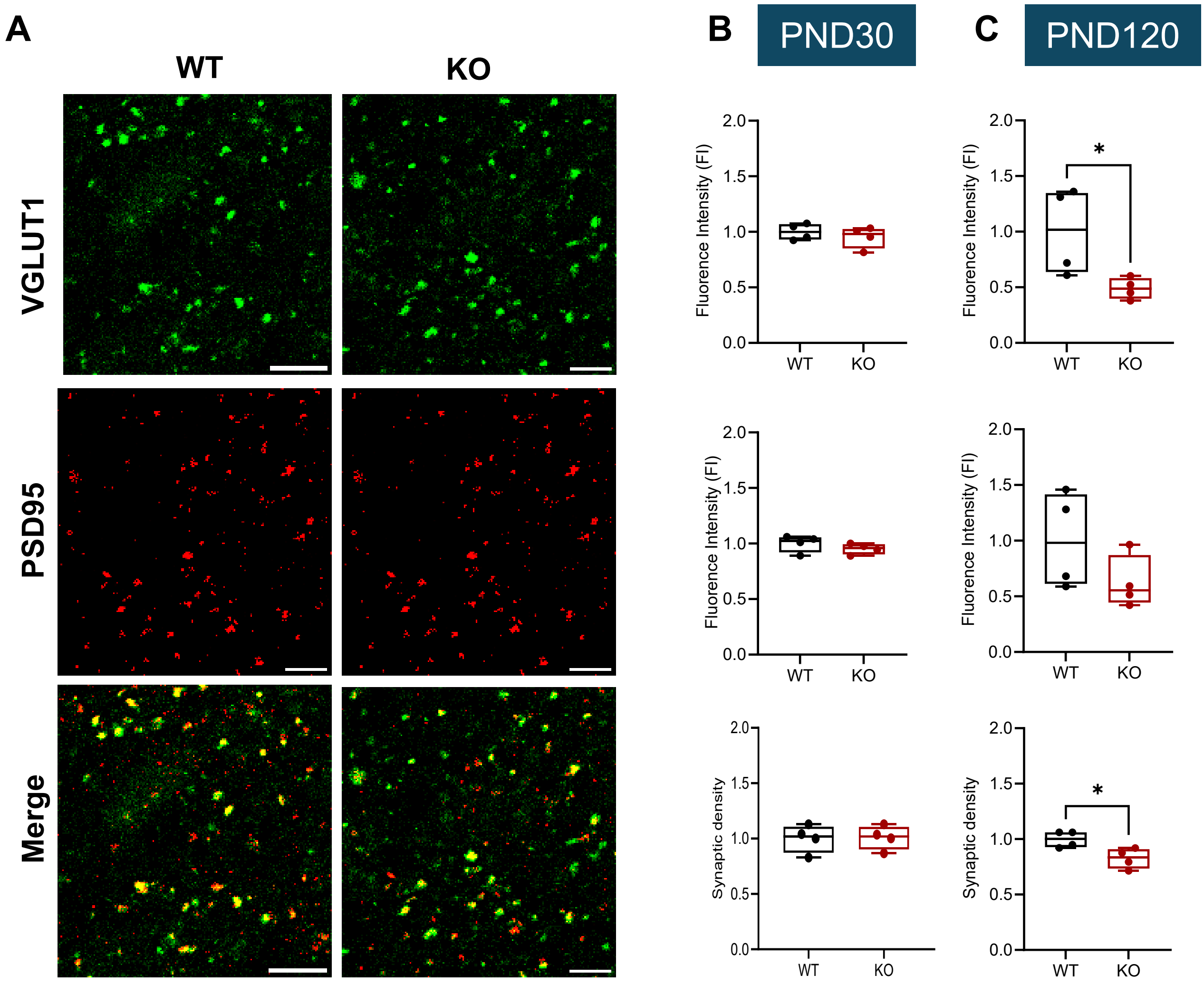

Supplementary Figure 6. The glutamatergic system of the LPAG is also altered in adult *Syn2KO* mice. (A) Representative images of LPAG from adult *Syn2* WT and KO mice acquired using a 63x oil immersion objective. Brain slices were immunostained for vGLUT and PSD95, while the bottom row displays the colocalization of the two signals used to identify synaptic contacts. Scale bars, 10 µm. **(C,D)** Box plots show the quantification of fluorescence intensity for vGLUT, PSD95, and the density of excitatory synapses (#synapses/µm²). Analyses were conducted on slices from young (PND30; **C**) and adult (PND120; **C**) mice.

Statistics. Box plots show median (center line), 25th-75th percentiles (box), and min-max values (whiskers). *p<0.05 (N = 4 WT and 4 KO for both time points). **(C)** Mann-Whitney's *U*-test. vGLUT1: U = 6, p=0.6857; PSD95: U = 3, p=0.200; Synaptic density: U = 8, p>0.999. **(D)** Mann-Whitney's *U*-test: vGLUT1, U = 0, p=0.028; PSD95: U = 3, p=0.20; Synaptic density: U = 0, p=0.029.

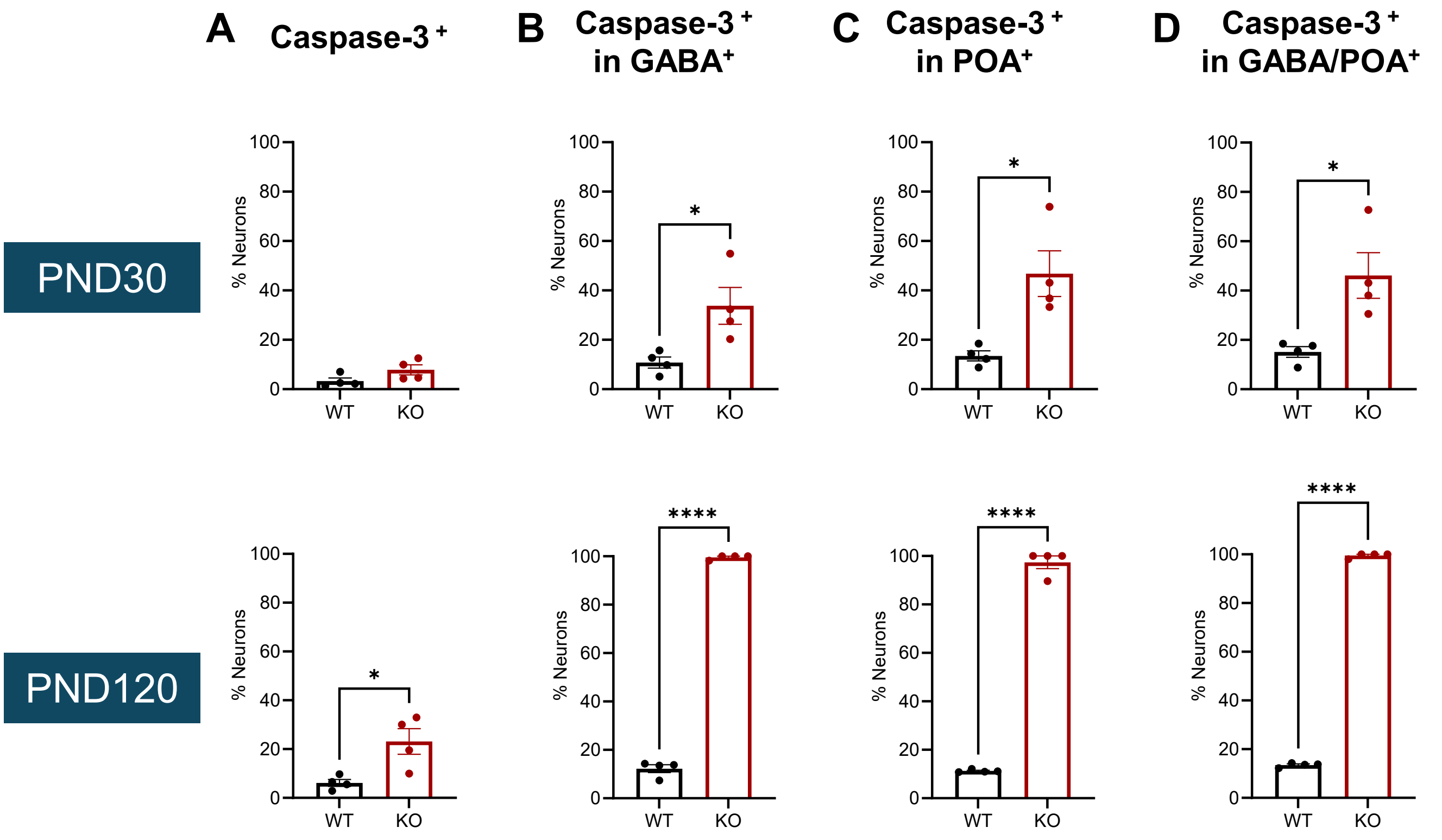

Supplementary Figure 7. The latent period of neuronal degeneration is present in different neuronal subpopulations of the LPAG network. Activated Caspase-3 signal detected at PND30 and PND120 in LPAG **(A)**, $GABA^+$ neurons of the LPAG **(B)**, neurons receiving input from the POA (POA^+, **C),** and $GABA^+$ neurons receiving input from the POA **(D)**.

Statistics. Data are expressed as means ± SEM. *p<0.05; ****p<0.0001 (N = 4 WT and 4 KO for both time points). **(A)** Unpaired Student's *t*-test. Young: $t(6) = 1.897$, p=0.11, Cohen's d=0.61; Adult: $t(6) = 3.121$, p=0.021, Cohen's d=0.79. **(B)** Young: $t(6) = 2.949$, p=0.026, Cohen's d=0.77; Adult: $t(6) = 51.65$, p<0.0001, Cohen's d=0.99. **(C)** Young: $t(6) = 3.513$, p=0.013, Cohen's d=0.82; Adult, $t(6) = 33.09$, p<0.0001, Cohen's d=0.99. **(D)** Young: $t(6) = 3.264$, p=0.0172, Cohen's d=0.80; Adult, $t(6) = 130.7$, p<0.0001, Cohen's d=1.

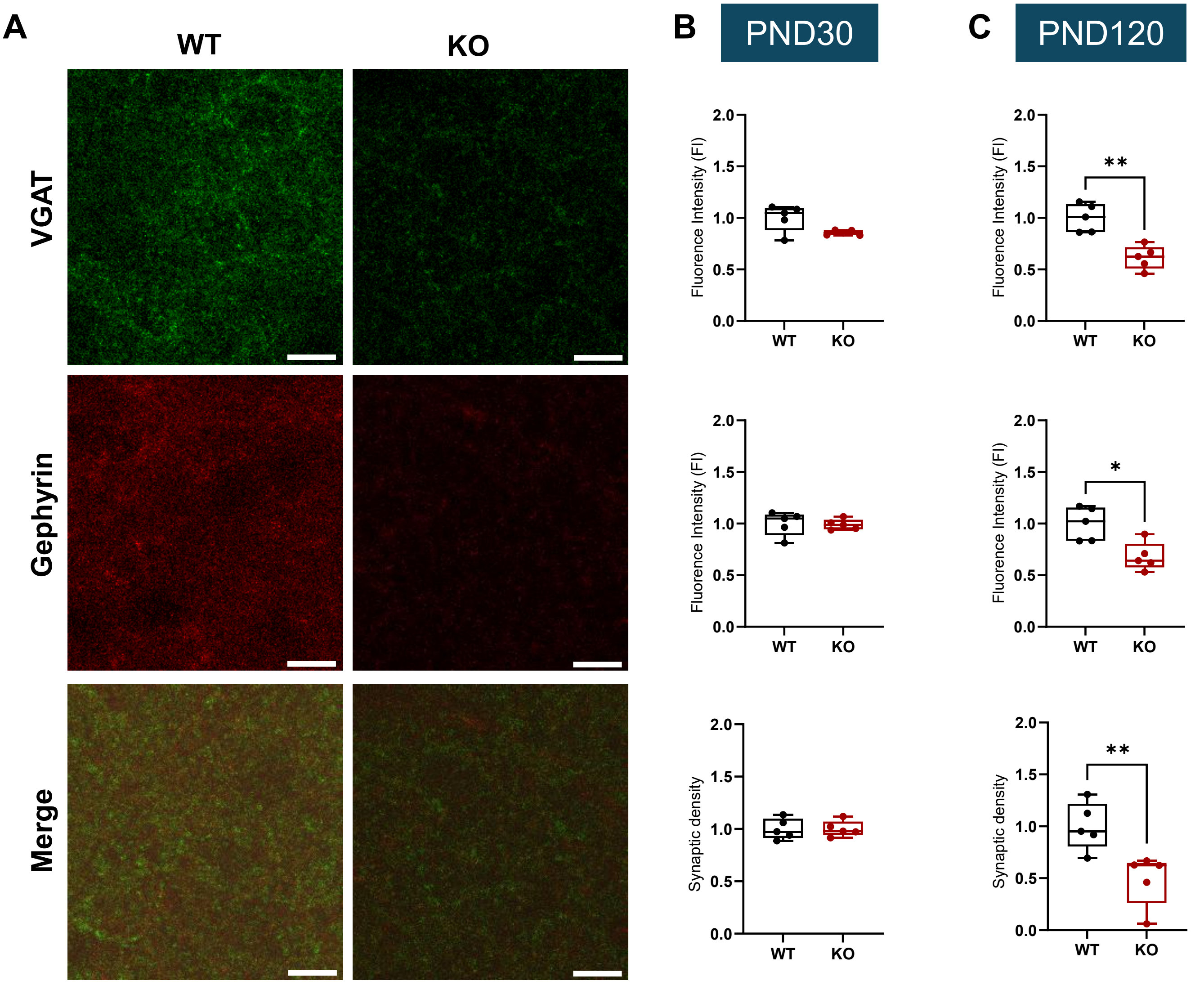

Supplementary Figure 8. Reduction of inhibitory synapses in the PPC parallels the loss of sensory integration in adult *Syn2KO* male mice. (A) Representative confocal images of PPC from adult WT and *Syn2KO* mice acquired using a 63x oil immersion objective. Brain slices were immunostained for vGAT and Gephyrin; the bottom row displays the colocalization of the two signals used to identify synaptic contacts. Scale bars, 10 µm. **(B,C)** Box plots show the quantification of the normalized fluorescence intensity for vGAT, Gephyrin, and density of inhibitory synapses (#synapses/µm²). Analyses were conducted on slices from young (PND30; **B**) and adult (PND120; **C**) mice.

Statistics. Box plots show median (center line), 25th-75th percentiles (box), and min-max values (whiskers). *p<0.05; **p<0.001 (N = 5 WT and 5 KO for both time points). **(B)** Mann-Whitney's *U*-test. vGAT: U = 5, p=0.151; Gephyrin: U = 9, p=0.55; Synaptic density: : U=12, p>0.9999. **(C)** Mann-Whitney's *U*-test. vGAT: U=0, p=0.0079; Gephyrin: U=2, p=0.032; Synaptic density: : U=0, p=0.008.

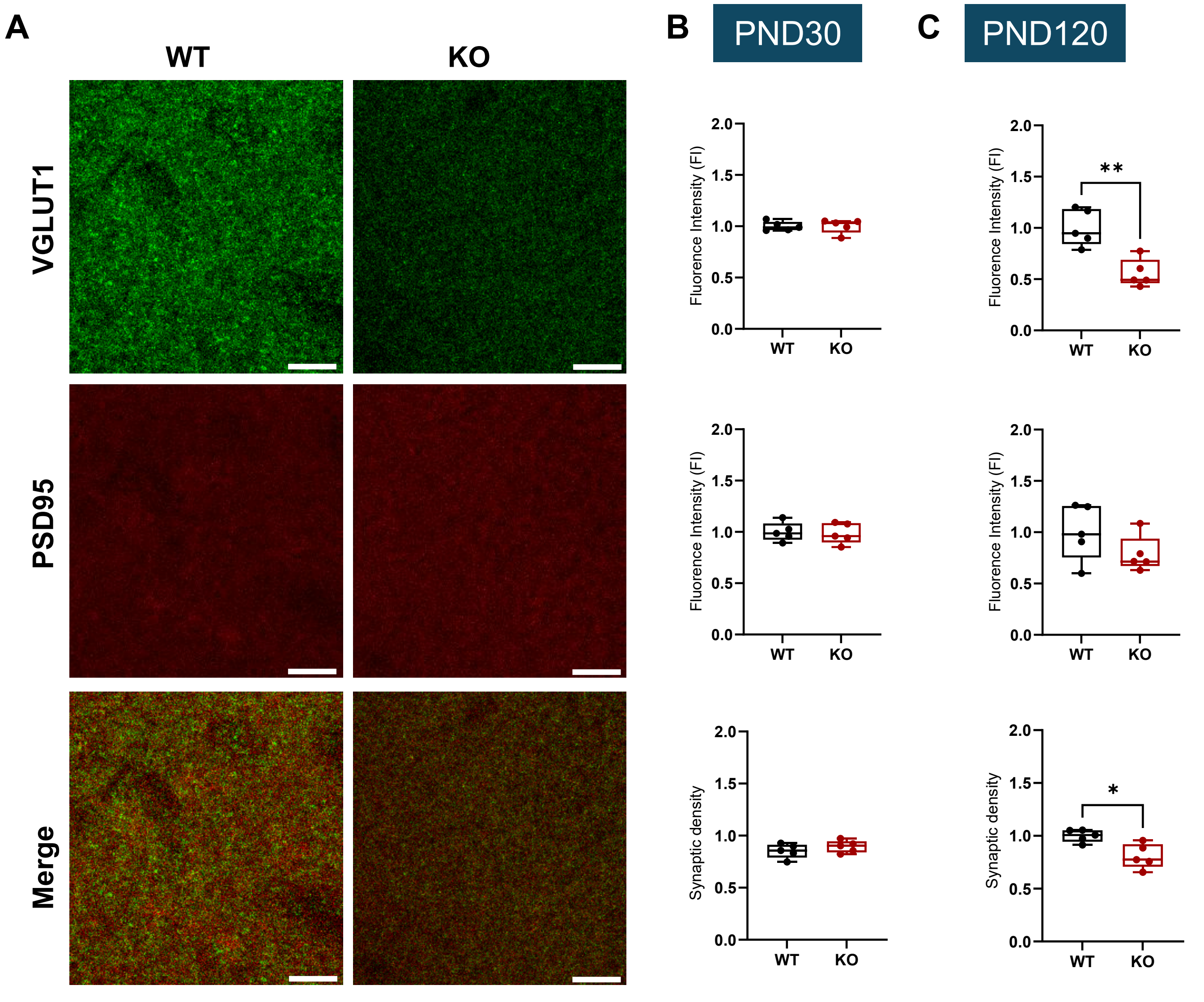

Supplementary Figure 9. The glutamatergic system in the PPC is altered in adult *Syn2KO male* mice. (A) Representative images of PPC from adult WT and *Syn2KO* mice acquired using a 63x oil immersion objective. Brain slices were immunostained for vGLUT1 and PSD95; the bottom row displays the colocalization of the two signals used to identify synaptic contacts. Scale bars, 10 µm. **(B,C)** Box plots show the quantification of the normalized fluorescence intensity for vGLUT1, PSD95, and density of excitatory synapses (#synapses/µm^2). Analyses were conducted on slices from young (PND30; **B**) and adult (PND120; **C**) mice.

Statistics. Box plots show median (center line), 25th-75th percentiles (box), and min-max values (whiskers). *p<0.05; **p<0.05 (N = 5 WT and 5 KO for both time points). (**B**) Mann-Whitney's *U*-test. vGLUT1: U=10, p=0.69; PSD95, U=11, p=0.84; Synaptic density: U=9, p=0.55. (**C**) Mann-Whitney's *U*-test. vGLUT1, U=0, p=0.0079; PSD95: U=7, p=0.31; Synaptic density: U=1, p=0.016.

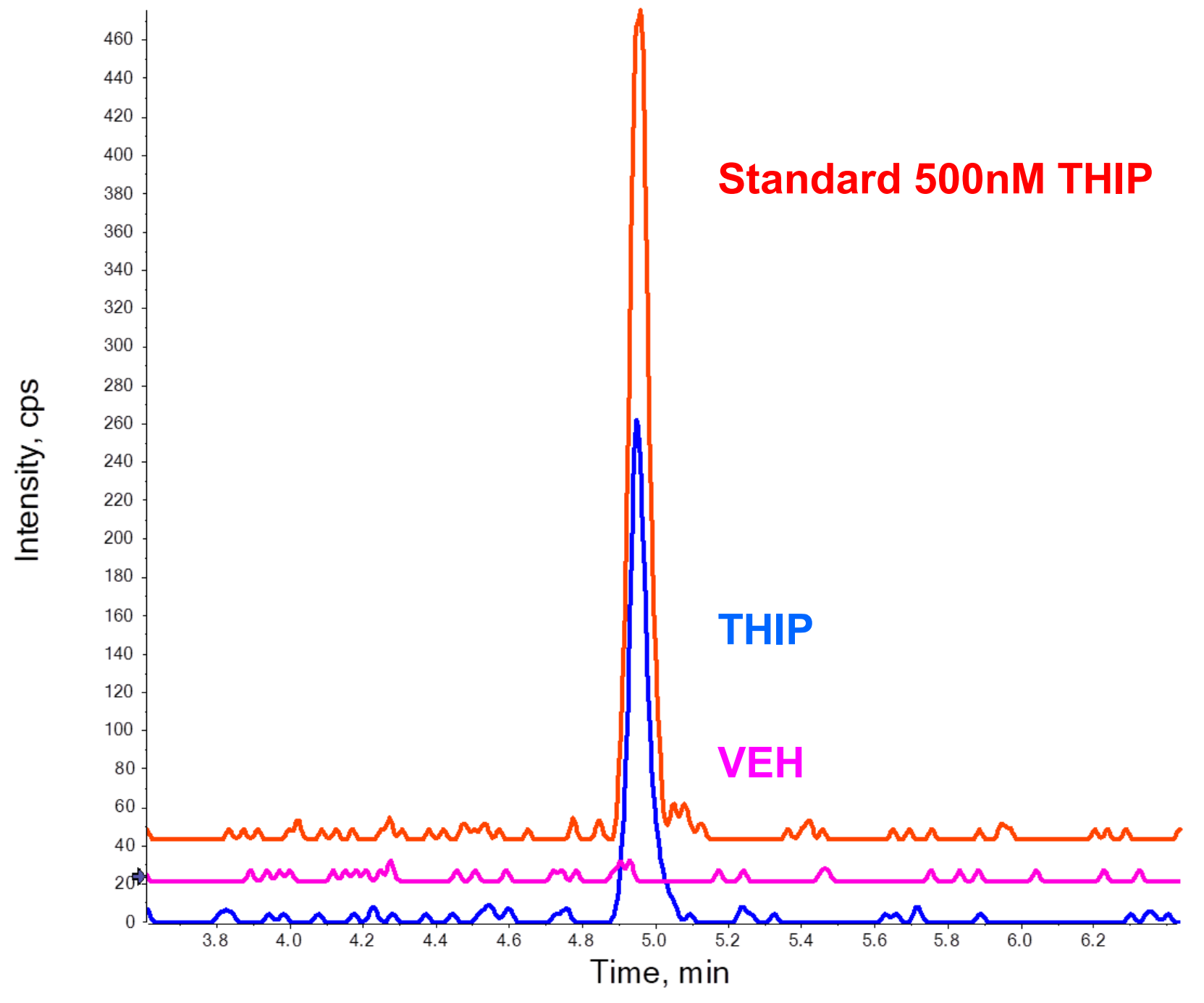

Supplementary Figure 10. LC-MS detection of THIP in the pup plasma. THIP was exclusively detected in the plasma of mice born from dams treated with THIP. The figure shows the LC-MS/MS traces of representative plasma samples from pups milked by vehicle-treated (VEH) and THIP-treated dams (THIP), overlapped with the trace of an authentic analytical THIP standard (500 nM).

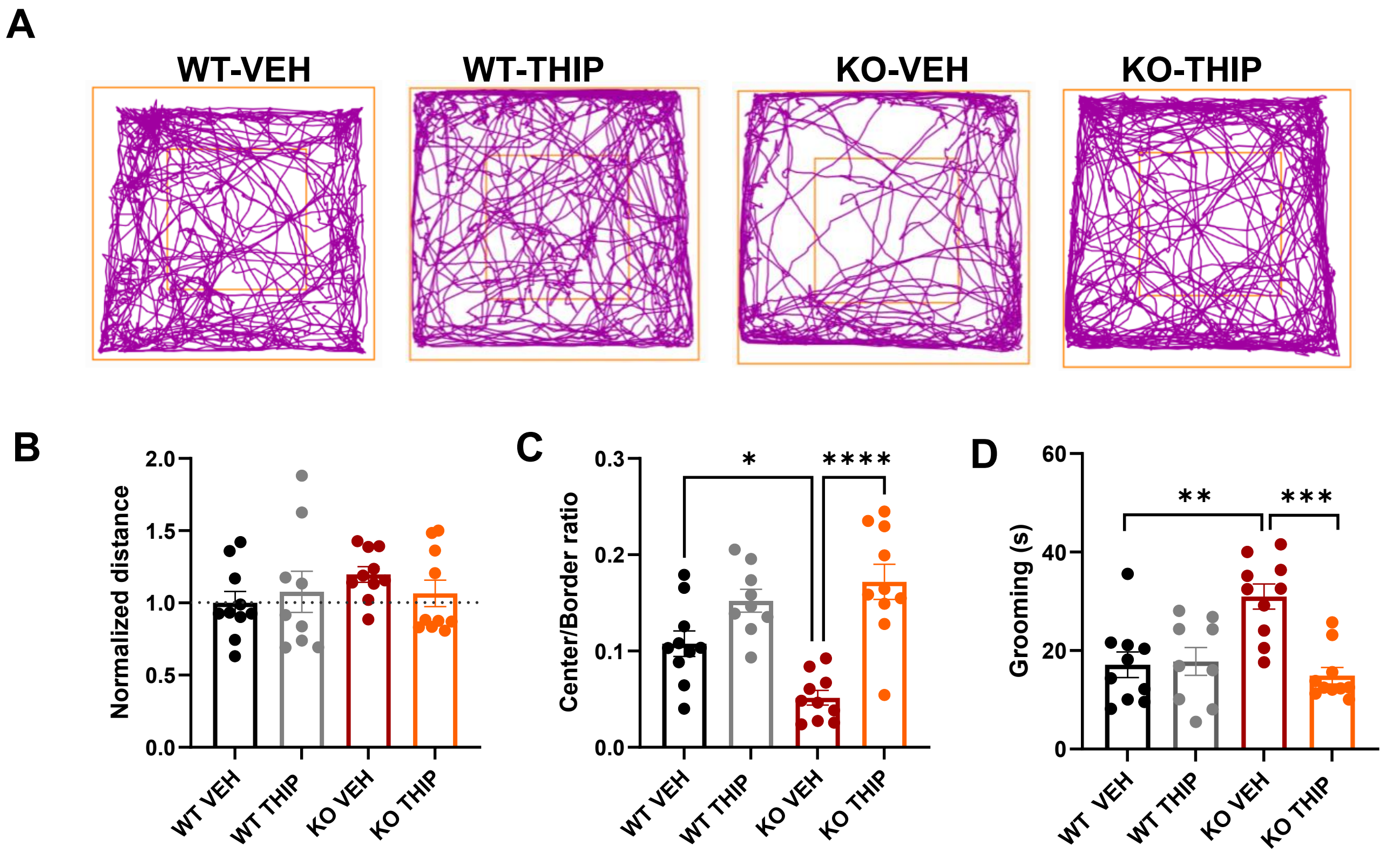

Supplementary Figure 11. THIP effects on anxiety profile and grooming behavior in adult *Syn2KO* male mice. (A) Representative tracking of locomotor activity performed by WT and KO male mice treated with vehicle or THIP during a 10-min open field test. **(B,C)** Box plots of the distance travelled (**B**), and the time spent in the borders *versus* the center of the arena (**C**). **(D)** Box plot of the time spent in grooming behavior.

Statistics. Data are expressed as means ± SEM with superimposed individual experimental points (N = 10 WT-VEH, 9 WT-THIP, 10 KO-VEH, 10 KO-THIP). *p<0.05; ***p<0.001, ****p <0.0001 (**B**) One-way ANOVA: $F_{3,35} = 0.78$, p=0.516, $\eta^2 = 0.062$. (**C**) One-way ANOVA: $F_{3,35} = 16.19$, p<0.0001, $\eta^2 = 0.581$ - Bonferroni's test: WT-VEH *vs* KO-VEH, p=0.028; KO-VEH vs KO-THIP p<0.0001. **(D)** One-way ANOVA: $F_{3,35} = 9.15$, p=0.0001, $\eta^2 = 0.440$ - Bonferroni's test: WT-VEH *vs* KO-VEH, p=0.0015; KO-VEH *vs* KO-THIP, p<0.002)

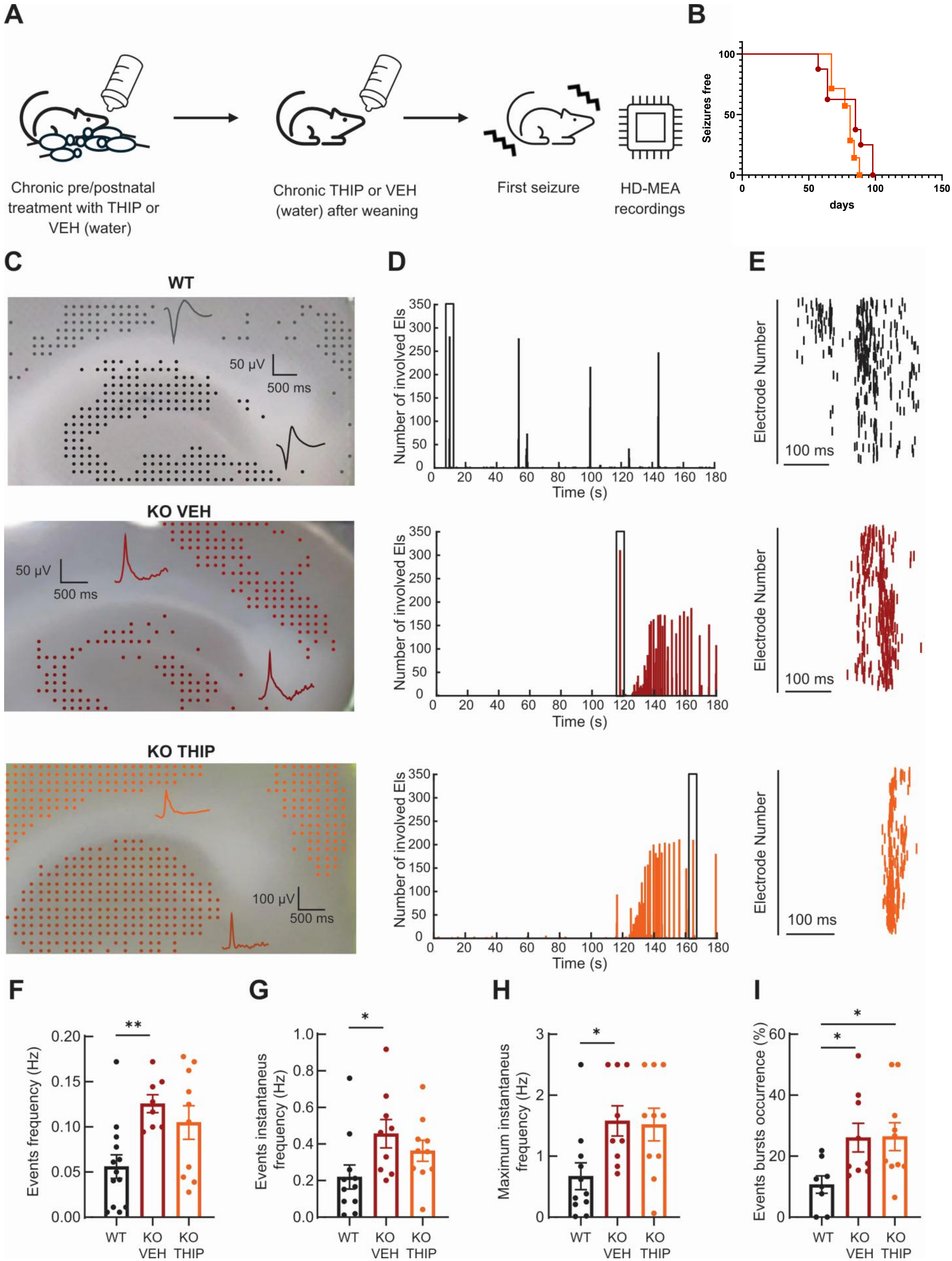

A
Chronic pre/postnatal treatment with THIP or VEH (water)
Chronic THIP or VEH (water) after weaning
First seizure
HD-MEA recordings
B
Seizures free
days
C
WT
50 µV
500 ms
KO VEH
50 µV
500 ms
KO THIP
100 µV
500 ms
D
Number of involved Els
Time (s)
E
Electrode Number
100 ms
F
Events frequency (Hz)
**
WT
KO VEH
KO THIP
G
Events instantaneus frequency (Hz)
*
H
Maximum instantaneus frequency (Hz)
*
I
Events bursts occurrence (%)
*
*

Supplementary Figure 12. THIP treatment does not suppress the epileptic phenotype in adult *Syn2KO* male mice (A) Experimental design. **(B)** Kaplan-Meier plot showing the percent of seizure-free *Syn2KO* male mice as a function of age. **(C)** Representative images of brain sections positioned on the HD-MEA chip, overlaid with the locations of electrodes where 4-AP (400 μM)-induced interictal events were detected. Interictal activity was observed in both hippocampal and cortical regions under all conditions: WT (blue, hippocampal event), vehicle-treated *Syn2KO* (KO, red, cortical event), and THIP-treated *Syn2KO* (KO+THIP, green, cortical event). Insets display representative averaged interictal waveforms for each condition. **(D)** Representative time histograms illustrating interictal event detection across all electrodes in a WT mouse (top), a KO mouse (middle), and a KO mouse treated with THIP (bottom). **(E)** Enlarged views of the black rectangles in D show the temporal distribution of interictal events across the active electrodes. **(F-I)** Quantification of interictal event properties, including average frequency (**F**), average instantaneous frequency (**G**), maximum instantaneous frequency (**H**), and the percentage of events occurring in bursts relative to the total number of detected events (**I**).
Statistics. (**B**) Kaplan-Meier, Long Rank (Mantel-Cox) test: p=0.1778 (N = 8 KO VEH and 8 KO THIP). **(F-I)** *p<0.05, **p<0.01; Kruskal-Wallis ANOVA with *post-hoc* Dunn's test (N = 11 WT, 9 KO, and 9 KO-THIP slices obtained from 3 mice per group).

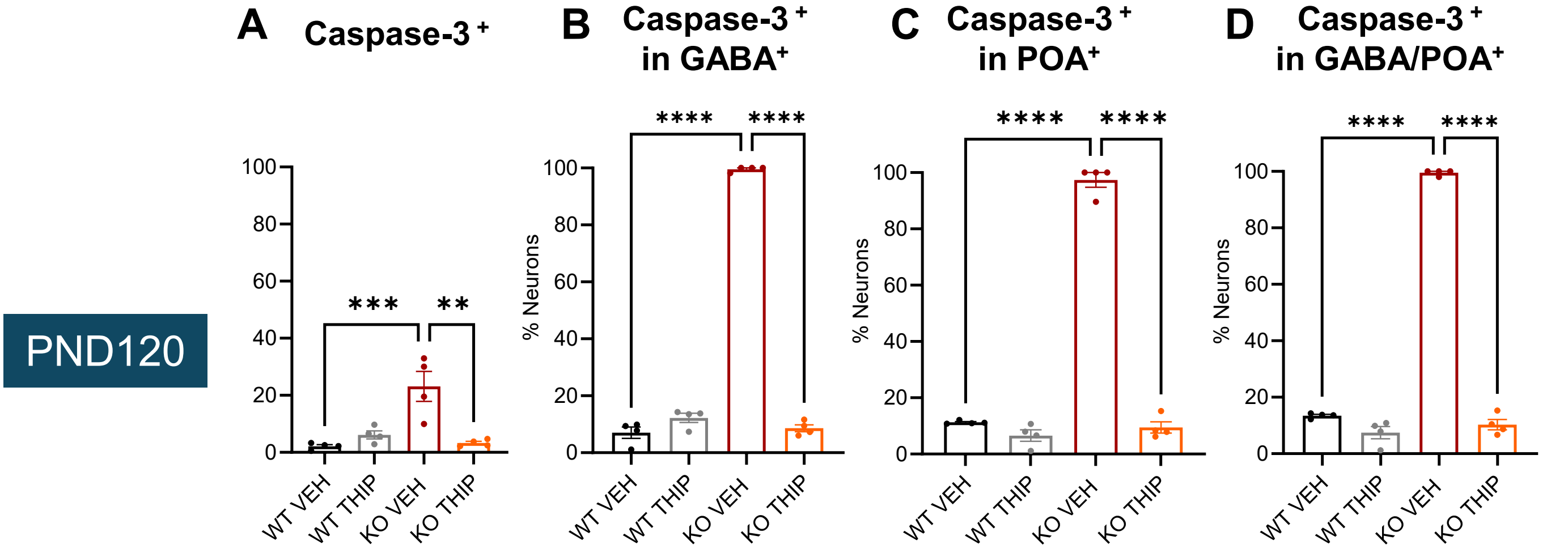

Supplementary Figure 13. Chronic THIP treatment counteracts neuronal degeneration across different neuronal populations in the LPAG of adult male *Syn2KO* mice. Caspase-3 activation was detected in LPAG **(A)**, GABA^{+} neurons of the LPAG **(B)**, neurons receiving input from the POA **(C),** and GABAergic neurons receiving input from the POA **(D)** of adult WT and *Syn2KO* male mice treated with either vehicle or THIP.

Statistics. Data are expressed as means ± SEM. **$p<0.01$; ***$p<0.001$; ****$p<0.0001$ (N = 4 WT and 4 KO). **(A)** One-way ANOVA: $F_{3,12} = 12.63$, p=0.0005, $\eta p^2 = 0.7595$ - Tukey's test: WT-VEH *vs* KO-VEH, p=0.008; KO-VEH *vs* KO-THIP, p=0.0013. **(B)** One-way ANOVA: $F_{3,12} = 965.0$, $p<0.0001$, $\eta p^2 = 0.9959$ - Tukey's test: WT-VEH *vs* KO-VEH, $p<0.0001$; KO-VEH *vs* KO-THIP LL, $p<0.0001$. **(C)** One-way ANOVA: $F_{3,12} = 525.8$, $p<0.0001$, $\eta p^2 = 0.9924$ - Tukey's test: WT-VEH *vs* KO-VEH, $p<0.0001$; KO-VEH *vs* KO-THIP, $p<0.0001$. **(D)** One-way ANOVA: $F_{3,12} = 931.6$, $p<0.0001$, $\eta p^2 = 0.9957$ - Tukey's test: WT-VEH *vs* KO-VEH, $p<0.0001$; KO-VEH *vs* KO-THIP LL, $p<0.0001$).

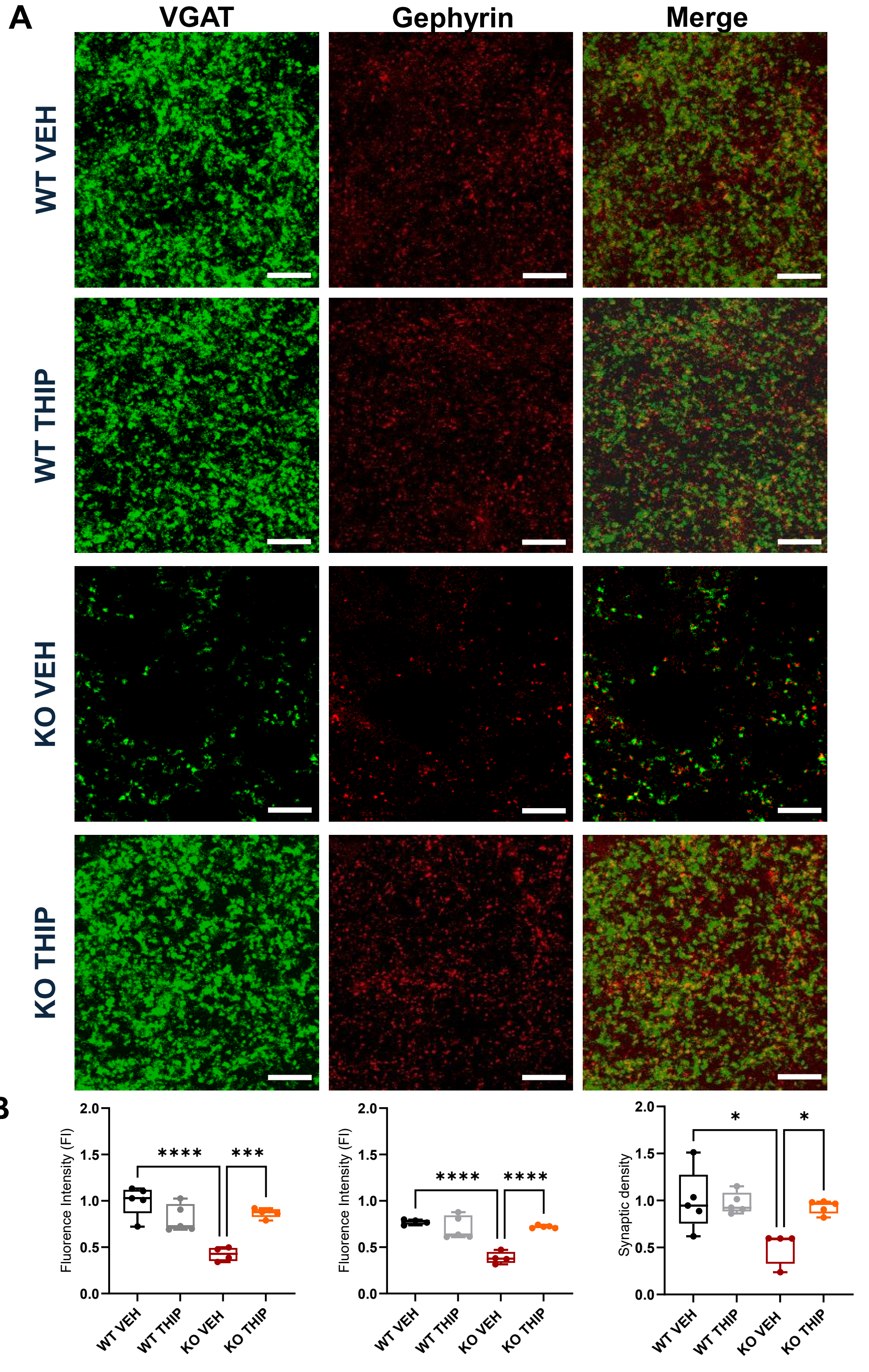

Supplementary Figure 14. Early restoration of GABAergic tonic current prevents the reduction of inhibitory synapses in the LPAG of adult *Syn2KO* male mice. (A) Representative confocal images of LPAG acquired using a 63x oil immersion objective from adult WT and KO mice treated with either VEH or THIP. Images represent brain slices stained for vGAT and Gephyrin; the bottom row displays the colocalization of the two signals used to identify synaptic contacts. Scale bars, 10 µm. **(B)** Box plots show the quantification of the normalized fluorescence intensity for vGAT, Gephyrin, and density of inhibitory synapses (#synapses/µm²).

Statistics. Box plots show median (center line), 25th-75th percentiles (box), and min-max values (whiskers). *$p<0.05$; ***$p<0.001$; ****$p<0.0001$ (N = 5 WT-VEH, 5 WT-THIP, 5 KO-VEH, 5 KO-THIP). **(B)** One-way ANOVA. vGAT: $F_{3,15} = 17.76$, $p<0.0001$, $\eta p^2 = 0.78$ - Tukey's test: WT-VEH *vs* KO-VEH, $p<0.0001$; KO-VEH *vs* KO-THIP, p= 0.0007; Gephyrin: $F_{3,14} = 22.61$, $p<0.0001$, $\eta p^2 = 0.83$ - Tukey's test: WT-VEH *vs* KO-VEH, $p<0.0001$; KO-VEH *vs* KO-THIP, $p<0.0001$); Synaptic density: $F_{3,15} = 5.76$, p = 0.0079, $\eta p^2 = 0.53$ - Tukey's test: WT-VEH vs KO-VEH, p=0.0151; KO-VEH vs KO-THIP, p=0.0337.

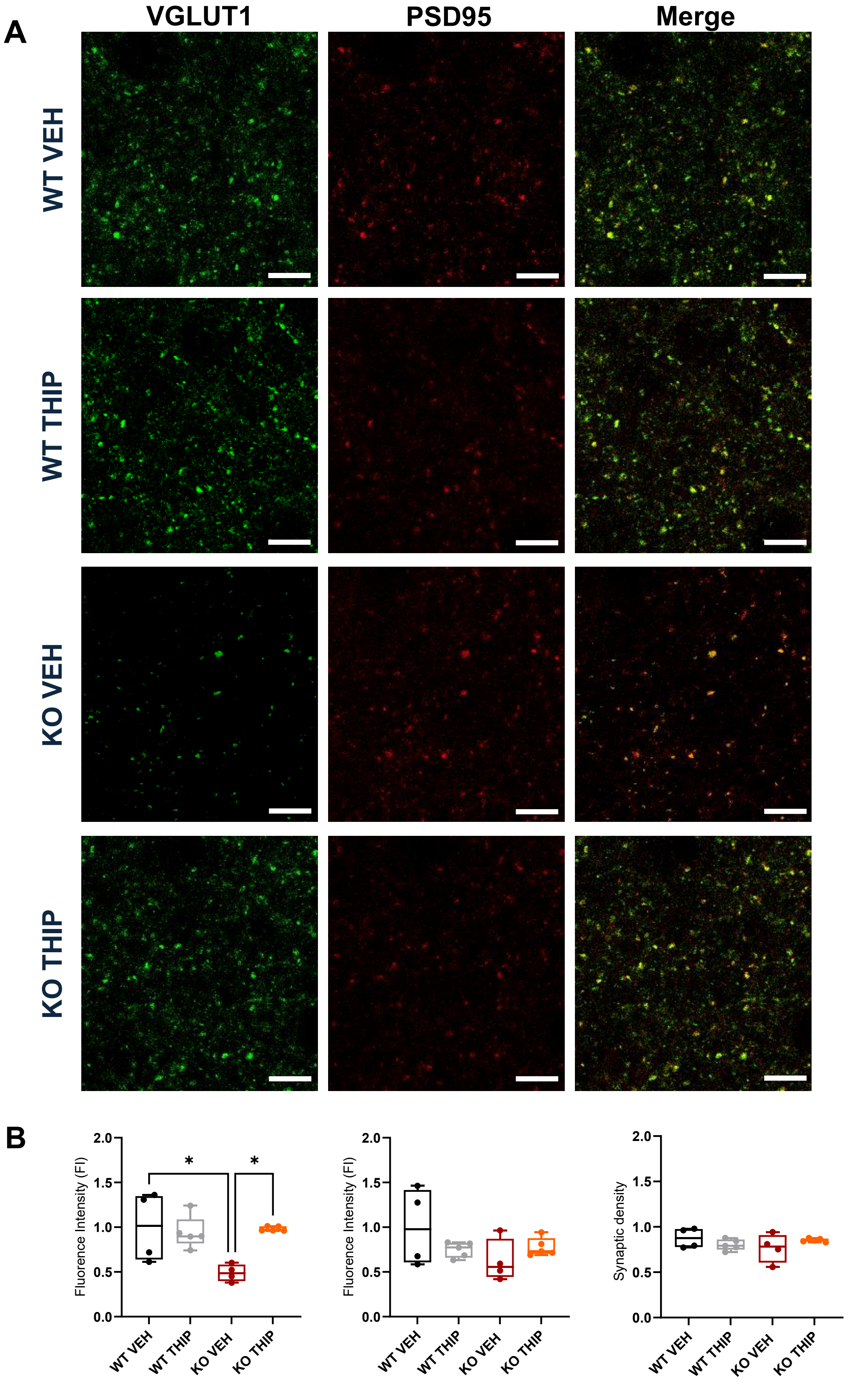

Supplementary Figure 15. Early restoration of GABAergic tonic current also restore the glutamatergic system in the LPAG of adult Syn2KO male mice. (A) Representative confocal images of LPAG acquired using a 63x oil immersion objective from adult WT and KO mice treated with either VEH or THIP. Images represent brain slices stained for vGLUT1 and PSD95; the bottom row displays the colocalization of the two signals used to identify synaptic contacts. Scale bars, 10 µm. **(B)** Box plots show the quantification of the normalized fluorescence intensity for vGLUT1, PSD95, and density of excitatory synapses (#synapses/µm²).

Statistics. Box plots show median (center line), 25th-75th percentiles (box), and min-max values (whiskers). *p<0.05 (N = 4 WT-VEH, 5 WT-THIP, 4 KO-VEH, 5 KO-THIP). **(B)** One-way ANOVA. vGLUT1: $F_{3,14} = 5.562$, p=0.010, ηp² = 0.54 - Tukey's test: WT-VEH *vs* KO-VEH, p=0.0461; KO-VEH *vs* KO-THIP, p=0.0498); PSD95: $F_{3,14} = 1.7$, p=0.21, ηp² = 0.2686; Synaptic density: $F_{3,14} = 1.140$, p=0.3671, ηp² = 0.1963.

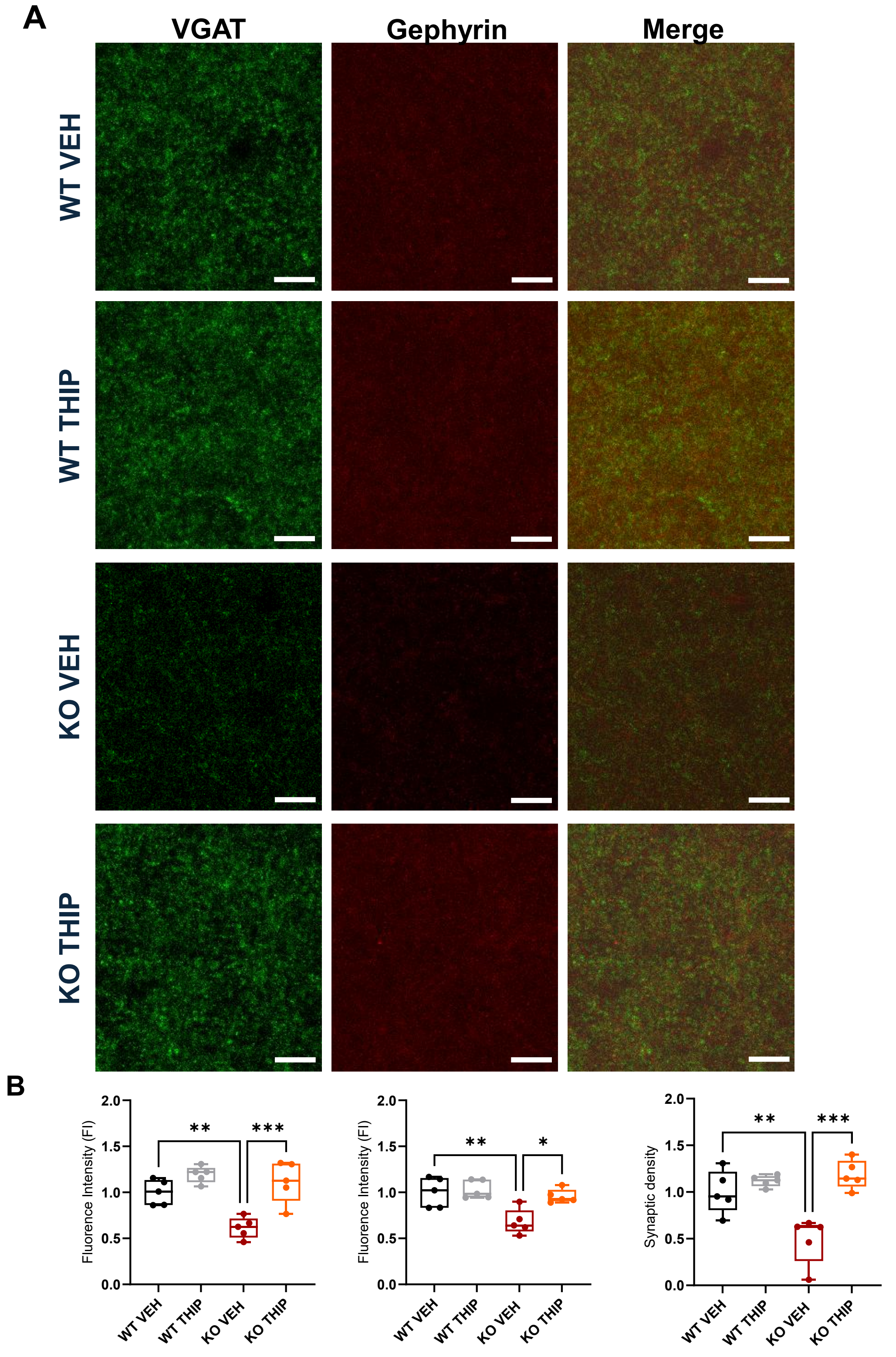

Supplementary Figure 16. Early restoration of GABAergic tonic current prevents the reduction of inhibitory synapses in the PPC of adult *Syn2KO* male mice. (A) Representative confocal images of PPC acquired using a 63x oil immersion objective from adult WT and KO mice treated with either VEH or THIP. Images represent brain slices stained for vGAT and Gephyrin; the bottom row displays the colocalization of the two signals used to identify synaptic contacts. Scale bars, 10 µm. **(B)** Box plots show the quantification of the normalized fluorescence intensity for vGAT, Gephyrin, and density of inhibitory synapses (#synapses/μm^2).

Statistics. Box plots show median (center line), 25th-75th percentiles (box), and min-max values (whiskers). *$p<0.05$; **$p<0.01$; ***$p<0.001$ (N = 5 WT-VEH, 5 WT-THIP, 5 KO-VEH, 5 KO-THIP). **(B)** One-way ANOVA. vGAT: $F_{3,16} = 14.51$, $p<0.0001$, $\eta p^2 = 0.7312$ - Tukey's test: WT-VEH *vs* KO-VEH, p=0.0047; KO-VEH *vs* KO-THIP, p=0.0004); Gephyrin: $F_{3,16} = 8.511$, p=0.0013, $\eta p^2 = 0.6148$ - Tukey's test: WT-VEH *vs* KO-VEH, p=0.0041; KO-VEH *vs* KO-THIP, p=0.012); Synaptic density: F*3,16* = 13.86, p=0.0001, $\eta p^2 = 0.7221$ - Tukey's test: WT-VEH *vs* KO-VEH, p=0.003; KO-VEH *vs* KO-THIP, p=0.0001.

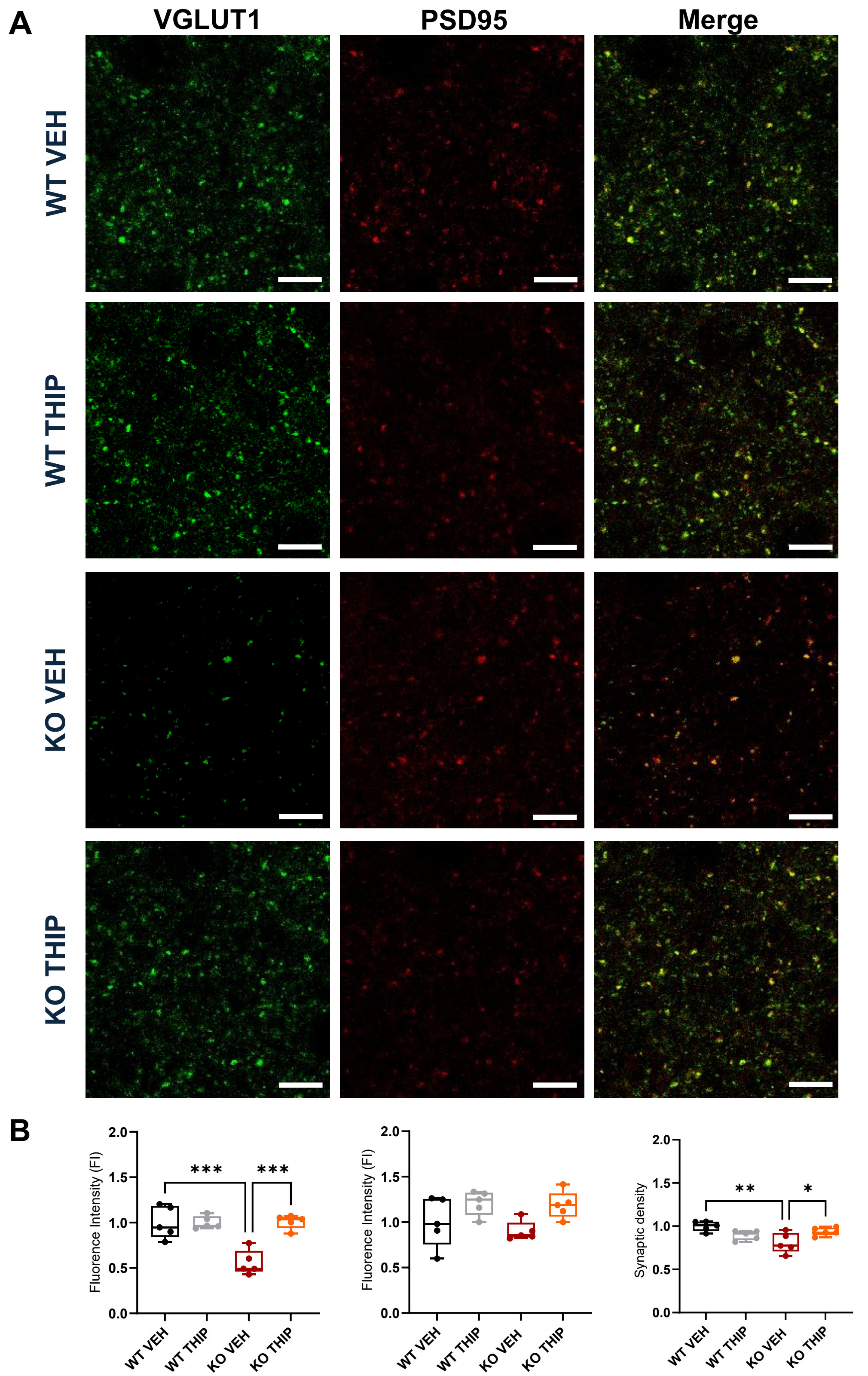

Supplementary Figure 17. Early restoration of GABAergic tonic current also restore the glutamatergic system in the PPC of adult Syn2KO male mice. . (A) Representative confocal images of PPC acquired using a 63x oil immersion objective from adult WT and KO mice treated with either VEH or THIP. Images represent brain slices stained for vGLUT1 and PSD95; the bottom row displays the colocalization of the two signals used to identify synaptic contacts. Scale bars, 10 µm. **(B)** Box plots show the quantification of the normalized fluorescence intensity for vGLUT1, PSD95, and density of excitatory synapses (#synapses/µm²). *Statistics*. Box plots show median (center line), 25th-75th percentiles (box), and min-max values (whiskers). *p <0.05; **p<0.01; ***p<0.001 (N = 5 WT-VEH, 5 WT-THIP, 5 KO-VEH, 5 KO-THIP). **(B)** One-way ANOVA. vGLUT1: $F_{3,16} = 16.29$, p<0.0001, $\eta p^2 = 0.7534$ - Tukey's test: WT-VEH *vs* KO-VEH, p=0.0002; KO-VEH *vs* KO-THIP, p=0.0001); PSD95: $F_{3,16} = 3.497$, p=0.0401, $\eta p^2 = 0.3960$ - Tukey's test: no significant differences between pairwise comparisons; Synaptic density: $F_{3,16} = 5.914$, p=0.0065, $\eta p^2 = 0.5258$ - Tukey's test: WT-VEH *vs* KO-VEH, p=0.0042; KO-VEH *vs* KO-THIP, p=0.05.
